# Grapevine nursery propagation material as source of fungal trunk disease pathogens in Uruguay

**DOI:** 10.3389/ffunb.2022.958466

**Published:** 2022-07-22

**Authors:** María Julia Carbone, Matías Gelabert, Victoria Moreira, Pedro Mondino, Sandra Alaniz

**Affiliations:** Departamento de Protección Vegetal, Facultad de Agronomía, Universidad de la República, Montevideo, Uruguay

**Keywords:** *Vitis vinifera*, propagation material, black-foot disease, petri disease, Diaporthe dieback, Botryosphaeria dieback

## Abstract

Grapevine fungal trunk diseases (GTDs) have become a serious problem for grapevines worldwide. Nursery vines infected during the propagation process are considered one of the main ways of dissemination of GTD pathogens. In this study, we examined the status of GTDs in grapevine planting material, from rootstocks and scion mother cuttings to grafted rooted vines ready to plant, according to the local nursery propagation process. During 2018-2019, internal symptoms of GTDs were examined in 2400 propagation materials and fungal isolations were carried out from a subsample of 1026 selected materials. Our results revealed that nursery grapevine plants produced in Uruguay have a high incidence of GTDs, regardless of the scion/rootstock combination. Typical brown to black streaks and sectorial wood necrosis were observed in materials on all propagation stages, with a markedly increasing incidence throughout the nursery process, reaching almost 100% in grafted rooted vines ready to plant. Botryosphaeria dieback, Petri disease and black-foot disease were the main GTDs found. The results showed that Botryosphaeria dieback and Petri disease pathogens infect materials from the early stages of the process, with a marked increase towards the end of the plant production process, whereas black-foot disease pathogens were found exclusively in vines ready to plant. Diaporthe dieback pathogens were also detected in materials in all stages but in a low proportion (less than 10% of infected material). Based on single locus analysis, the 180 isolates selected were placed into eight genera and 89% identified within 22 fungal species associated with GTDs, with *Phaeoacremonium oleae* and *Diaporthe terebinthifolii* as new records on grapevine worldwide. Our results have concluded that locally produced vines are one of the main ways of dissemination of GTD pathogens and showed that a nursery sanitation programme is required to reduce the incidence of these diseases.

## Introduction

Grapevine fungal trunk diseases (GTDs) have become a serious problem for grapevine growers around the world (Martin and Cobos, 2007; Romanazzi et al., 2009; Bruez et al., 2013; Yan et al., 2013; Ridgway et al., 2014; Úrbez-Torres et al., 2014). The significant increment of decline symptoms in grapevines during the last decades causes loss of productivity and reduction in longevity, especially in young vineyards (Gramaje and Armengol, 2011). This situation makes growers replant vast areas, resulting in important economic losses in viticulture worldwide (Gramaje et al., 2018). External decline symptoms on young vineyards include reduced vigour, retarded or absent sprouting, shortened internodes, reduced foliage, chlorotic foliage with necrotic margins, reduced leaf size, failure of the graft unions, wilting and often leading to death of affected plants (Scheck et al., 1998a; Rego et al., 2000; Fourie and Halleen, 2001).

Petri disease (PD), caused mostly by *Phaeomoniella chlamydospora, Cadophora luteo-olivacea* and numerous species of *Phaeoacremonium* (Scheck et al., 1998b; Mugnai et al., 1999; Crous and Gams, 2000; Mostert et al., 2006a; Halleen et al., 2007; Gramaje et al., 2011; Navarrete et al., 2011), and black-foot disease (BFD), caused by species of *Cylindrocarpon*-like asexual morphs, *Campylocarpon* and *Cylindrocladiella* genera (Mohammadi et al., 2009; Agustí-Brisach and Armengol, 2013; Lombard et al., 2014; Carlucci et al., 2017) are the main GTDs associated with young decline symptoms (Rego et al., 2000; Gramaje and Armengol, 2011; Carlucci et al., 2017; Gramaje et al., 2018).

In addition, Botryosphaeria dieback (BD) caused by species belonging to *Botryosphaeriaceae*, as well as, though less frequently, Diaporthe dieback (DD) caused by species of *Diaporthe*, have been isolated from young vines showing decline symptoms (Giménez-Jaime et al., 2006; Úrbez-Torres et al., 2006; Martin and Cobos, 2007; Moreno-Sanz et al., 2013; Whitelaw-Weckert et al., 2013; Larignon et al., 2015; Carlucci et al., 2017).

External symptoms do not allow an easy distinction among GTDs because they can overlap (Gramaje et al., 2018; Mondello et al., 2018). However, internally, vines affected by PD show black discoloration of the xylem vessels because of the accumulation of phenolic compounds. These discolorations are seen as dark brown to black spots and dark streaks, in cross and longitudinal section, respectively (Mugnai et al., 1999; Mostert et al., 2006a). Vines affected by BFD present brown to dark streaks that develop from the base of the rootstock, wood necrosis at the base of the trunk, sunken necrotic root lesions and reduced root biomass (Rego et al., 2000; Halleen et al., 2006; Alaniz et al., 2007). Internal symptoms of Botryosphaeria dieback include typical trunk cankers or sectorial wood necrosis as well as vascular streaking (Larignon and Dubos, 2001; Phillips, 2002; van Niekerk et al., 2004), symptoms which are also observed in DD infected vines (Úrbez-Torres et al., 2013; Guarnaccia et al., 2018).

Many studies have highlighted the role of infected propagation material as a major source of spread of GTD pathogens (Gramaje and Armengol, 2011). Several researchers have concluded that a high percentage of the plants ready to plant are infected by PD, BFD, BD and DD fungal pathogens, acting alone or simultaneously, even when plants are externally apparently healthy (Halleen et al., 2003; Aroca et al., 2006; Giménez-Jaime et al., 2006; Halleen et al., 2006; Rego et al., 2009; Spagnolo et al., 2011; Agustí-Brisach et al., 2013a; Carlucci et al., 2017; Guarnaccia et al., 2018; Pintos et al., 2018; Berlanas et al., 2020; Maldonado-González et al., 2020).

Traditional grapevine propagation techniques were analysed and described by Gramaje and Armengol (2011). Briefly, dormant cuttings are taken from rootstock and scion mother vines for bench grafting, rooting or field budding. The propagation process includes cold storage, hydration, disbudding, grafting, callusing, and rooting of grafted plants in the nursery field (Gramaje and Armengol, 2011). Most of these steps have been identified as an opportunity for GTD pathogens to cause new infections. Firstly, several authors have reported that rootstocks and scion mother used to propagate vines are infected by PD, BFD, BD and DD pathogens with varying incidence, and constitute a primary inoculum source for GTD pathogens (Rego et al., 2001; Fourie and Halleen, 2002; Edwards and Pascoe, 2004; Fourie and Halleen, 2004; Retief et al., 2006; Whiteman et al., 2007; Zanzotto et al., 2007; Aroca et al., 2010; Serra et al., 2011; Billones-Baaijens et al., 2013). Additionally, GTD pathogens have been detected in water of hydration tanks, washings of scissors used in the grafting process, washings of grafting machines, and in the peat used for the callusing stage (Retief et al., 2006; Aroca et al., 2010; Gramaje et al., 2011; Agustí-Brisach et al., 2013a). Finally, the nursery soil where the rooting phase of the grafted plants occurs, constitutes the main inoculum source of BFD pathogens, which are known to be soil-borne pathogens (Rego et al., 2001; Halleen et al., 2006; Agustí-Brisach et al., 2013b; Agustí-Brisach et al., 2014; Berlanas et al., 2017). Furthermore, the soil is also a reservoir for PD pathogens (Retief et al., 2006; Agustí-Brisach et al., 2013b).

In Uruguay, BFD and PD pathogens have been reported to cause decline symptoms in young vines (Abreo et al., 2010; Abreo et al., 2011). Also, the analysis of asymptomatic canes of rootstocks and scions mother plants detected the presence of *Ph. chlamydospora* and *Phaeoacremonium* spp., as well as *Botryosphaeriaceae* pathogens (Abreo et al., 2011; Abreo et al., 2013). However, to the best of our knowledge, the occurrence of infections of GTD pathogens during the propagation process and the health status of the nursery plants produced in local nurseries has not been extensively explored. Thus, the aim of this study was: 1) to identify the GTDs and associated pathogens affecting nursery grapevine plants produced in Uruguay and quantify their incidence and 2) to find out the steps in which the incidence of GTDs increases during the local nursery propagation process.

## Material and methods

### Sampling of grapevine propagation material

During 2018-2019, a total of 2400 grapevine propagation materials were sampled from the main grapevine commercial nursery in Uruguay, located in Las Violetas, Canelones (34°34′48.45″ S and 56°17′50.17″ W). Samples were taken randomly at the following four stages of the standard propagation process in the nursery: (1) rootstock and scion cuttings just after they were collected from mother plants in winter (1.1 m long), (2) rootstock and scion cuttings after storage during 2-3 months at 5-6°C and hydration for 24-48 hours in water before grafting (1.1 m long), (3) grafted plants after callusing stage (the callusing boxes contained water, according to standard practice in the nursery) and (4) dormant rooted grafted plants ready to plant. The rootstocks cuttings derived from nursery-grown mother plants, whereas scion cuttings were collected from commercial vineyards, according to the usual process implemented in the nursery. The rootstock and scion cultivars and rootstock-scion combinations sampled are listed in **Table 1**.

**Table 1:** Grapevine propagation materials sampled from four stages of the nursery process in 2018 and 2019: scion cultivars, rootstocks, and combination cultivar-rootstocks.

Grapevine propagation materials	2018		2019	
	Grapevine cultivar/Rootstock	Samples collected	Grapevine cultivar/Rootstock	Samples collected
***Stage 1-*** r ***ootstock and scion cuttings from mother plants***				
Rootstock	Gravesac, 1103P, SO4, 101-14, 3309C	250	Gravesac, SO4, 3309C	150
Scion	Albariño, Tannat, Marselan, Merlot, Lácrima-Christi	250	Albariño, Tannat, Marselan	150
***Stage 2-*** r ***ootstock and scion cuttings after cold storage and hydration***				
Rootstock	Gravesac, 1103P, SO4, 101-14, 3309C	250	Gravesac, SO4, 3309C	150
Scion	Albariño, Tannat, Marselan, Merlot, Lácrima-Christi	250	Albariño, Tannat, Marselan	150
***Stage 3- grafted plants after callusing***				
Cultivar/Rootstock	Albariño/Gravesac, Albariño/101-14, Lácrima Christi/SO4, Tannat/1103P, Moscatel de Hamburgo/SO4	250	Marselan/3309C, Tannat/Gravesac, Albariño/SO4	150
***Stage 4- rooted grafted plants***				
Cultivar/Rootstock	Merlot/101-14, Lácrima Christi/1103P, Cabernet Franc/3309C, Tannat/Gravesac, Chardonnay/SO4	250	Albariño/Gravesac, Albariño/101-14, Tannat/1103P	150

### Internal disease symptoms

The occurrence of internal symptoms resembling GTDs, such as dark spots or streaks and sectorial wood necrosis, was examined in all samples collected. Propagation materials in stages 1 and 2 were examined as follows: cross-sectional and longitudinal sections were made in the first 0.15 m from the bottom part of the cutting (the portion of the cutting nearest to the mother trunk), and at 0.95-1.10 m, the top of the cutting (the portion of the cutting furthest from the mother trunk). Callused grafted plants and rooted grafted plants, stages 3 and 4 respectively, were analysed performing cross and longitudinal cuts in the grafted union section including the scion, and at the basal part of the plant (=foot). Additionally, materials in stage 4 were examined in the middle part of the rootstock between the graft union and the foot. The incidence of internal wood symptoms was calculated for each portion of rootstock, scion cultivar and rootstock-scion combination evaluated.

### Fungal isolation and morphological identification

A subsample of 1026 materials was selected to perform fungal isolations. To do this, samples with typical internal symptoms of GTDs were picked up, but also asymptomatic samples were selected for isolations. All samples were first surface sterilized by soaking each portion in 95% ethanol for 1 s followed by flaming (Delgado et al., 2016). Then, the bark was removed with a sterile scalpel and seven thin pieces of wood, 0.5 cm long, of each section were taken from the margin between necrotic and apparently healthy wood tissue and plated onto potato dextrose agar (PDA) (Oxoid Ltd., Hampshire, England) supplemented with 0.4 g L^−1^ of streptomycin sulphate (Sigma-Aldrich, China). From the asymptomatic materials, pieces of wood were taken from each section at random and processed as indicated above. Additionally, root sections were included in the isolations from the basal part of the plants in stage 4.

Plates were incubated for 5 to 21 days at 25°C in darkness and were examined daily to check for fungal growth. Based on phenotypical characteristics such as growth rate, colour, texture and shape of colonies, and microscopic examination of fruiting structures, conidiophores, and conidia (Crous and Gams, 2000; van Niekerk et al., 2004; Mostert et al., 2006b; Chaverri et al., 2011; Agustí-Brisach and Armengol, 2013; Úrbez-Torres et al., 2013), cultures resembling species within GTD causal agents were selected and sub-cultured on PDA and incubated at the same conditions. Subsequently, isolates were sub-cultured by hyphal tipping on PDA to purify the culture (Úrbez-Torres et al., 2006) and then stored in colonized sterile filter papers at -20°C (Peever et al., 1999).

Based on the phenotypical characteristics indicated above, fungal isolates were grouped into four categories according to the GTDs expected to be associated with the grapevine propagation material: *i*) Petri disease (PD); *ii*) black-foot disease (BFD); *iii*) Botryosphaeria dieback (BD) and *iv*) Diaporthe dieback (DD). Petri disease fungi were further divided into *Phaeomoniella chlamydospora*-like isolates and *Phaeoacremonium* spp. isolates. To induce the production of reproductive structures, isolates belonging to *Botryosphaeriaceae* and *Diaporthe* genus were plated onto water agar with sterilized pine needles on the agar surface and onto PDA, respectively, and incubated under near-UV light with a 12-hr photoperiod at 25°C (Úrbez-Torres et al., 2006; Guarnaccia et al., 2018). A representative subsample of each group, attempting to include as much diversity as possible, was selected for subsequent molecular identification.

### Molecular identification

Total DNA was extracted from pure cultures grown on PDA at 25°C in darkness for 7 to 14 days using the commercial kit Quick-DNA™ Fungal/Bacterial Miniprep Kit (ZymoResearch, USA) following the manufacturer´s instructions. All DNA suspensions were stored at -20°C for further studies.

Sequences were generated from internal transcribed spacer region and 5.8S rRNA (ITS) with ITS1/ITS4 primers (White et al., 1990) for *Phaeomoniella chlamydospora*-like isolates, beta-tubulin (TUB2) with T1/BT2b primers (O’Donnell and Cigelnik, 1997) for *Phaeoacremonium* isolates, histone 3 (HIS3) with CYLH3F/CYLH3R primers (Crous et al., 2004) for BFD isolates and elongation factor 1-α (TEF) with EF1-728F/EF1-986R primers (Carbone and Kohn, 1999) for *Botryosphaeriaceae* and *Diaporthe* isolates. These loci were proposed to be the most informative for each pathogen group (Tegli et al., 2000; Cabral et al., 2012a; Phillips et al., 2013; Santos et al., 2017; Marin-Felix et al., 2019).

Polymerase chain reaction (PCR) amplifications were performed on a MultiGene™ Mini (Labnet International, Inc., USA). Each PCR reaction contained 1x PCR buffer, 2.5 mM MgCl2, 0.4 mM of each dNTP, 0.4 μM of each primer, 1 U of DNA polymerase (Bioron, Germany) and 1 μL of template DNA. The PCR reaction was adjusted to a final volume of 20 μl with MQ water. The PCR conditions consist of an initial step of 94°C for 3 min followed by 34 cycles for ITS, TUB2 and TEF regions and 40 cycles for HIS3 gene of denaturation at 94°C for 30 s, annealing at 57°C for ITS and TUB2 and 55°C for HIS3 and TEF for 30 s, and elongation at 72°C for 45 s. A final extension was performed at 72°C for 10 min. PCR products were analysed in 1.5% agarose gels stained with GelRed™ and visualized in a transilluminator under UV light. A GeneRuler 100-bp DNA ladder plus was used as a molecular weight marker (Thermo, Lithuania).

PCR products were purified and sequenced in Macrogen Inc., Seoul, Korea. Preliminary identifications were obtained by comparing the sequences with those deposited in the GenBank using the BLAST source (https://blast.ncbi.nlm.nih.gov/Blast.cgi). The BLAST identifications were confirmed by phylogenetic analysis using Bayesian inference (BI) and Maximum Likelihood (ML) methods. For this, alignments were constructed using ClustalW program, available within MEGA 11.0.11 program (https://www.megasoftware.net/). Alignments were manually edited when necessary. Related sequences as well as sequences of the phylogenetically closest species obtained from the GenBank, including ex-type isolates, were incorporated to the alignments (**Supplementary Table 1**). BI and ML analyses were inferred with MrBayes v3.2.7a and RAxML v8.2.12 programs, respectively, implemented in CIPRES Science Gateway v3.3 (http://www.phylo.org/). For BI analysis, best-fit models of nucleotide substitution were selected for each genus according to the Akaike information criterion (AIC), using the jModelTest2 v2.1.6 tool (Darriba et al., 2012) implemented in CIPRES Science Gateway v3.3 (**Supplementary Table 2**). Four Markov chain Monte Carlo (MCMC) chains were run simultaneously starting from a random tree to 10 million of generations. Trees were sampled every 1000 generations, and the first 2500 were discarded as the burn-in phase of each analysis. Posterior probabilities were determined from a majority-rule consensus tree generated from the remaining 7500 trees. For the ML analysis, generalized time-reversible with gamma correction (GTR + GAMMA) nucleotide substitution model and 1000 bootstrap iterations were indicated. The other parameters were used as default settings. The sequences generated in this study were deposited in GenBank database (**Supplementary Table 3**).

### Fungal trunk disease incidence

The GTD incidence, alone or in combination, was calculated based on the incidence of fungal pathogens in propagation materials at the four stages of the nursery process, for each portion of wood analysed. The incidence was determined as follows: (number of materials infected by each GTD or specific combination of GTDs, divided by the total number of materials processed) x 100.

## Results

### Internal disease symptoms

**Figure 1:**
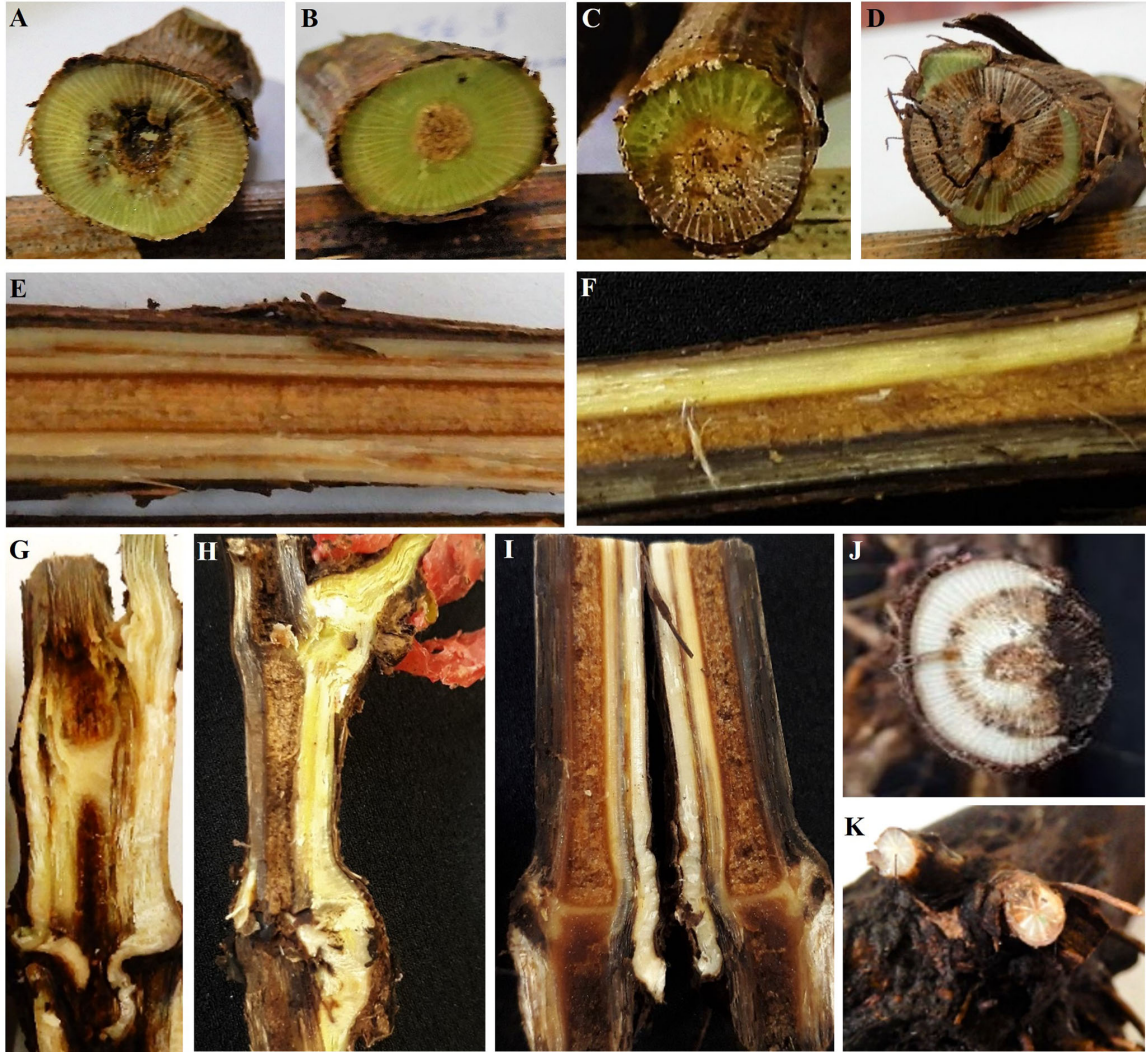
Internal symptoms caused by fungal pathogens associated with grapevine trunk diseases. Cross section of cuttings showing **(A, B)** black discoloration of the xylem vessels seen as black spots and **(C, D)** sectorial wood necrosis. Longitudinal section of cuttings showing **(E)** black discoloration of the xylem vessels seen as dark streaks and **(F)** sectorial wood necrosis. Longitudinal section of the graft union area showing **(G)** dark streaks and **(H)** sectorial wood necrosis. Longitudinal section of the base of the rootstock showing **(I)** dark streaks that develops from the base and sectorial wood necrosis. Cross section of the base of the rootstocks showing **(J)** a half-ring of black spots and sectorial wood necrosis. Cross section of roots showing **(K)** sectorial wood necrosis.

All grapevine cultivars and rootstocks presented typical internal wood symptoms of GTDs in the four stages of the nursery propagation process in both years of evaluation, except from the rootstock 101-14. This rootstock showed no internal wood symptoms in stages 1 and 2 in 2018 (the only year of evaluation of this rootstock). Around the 80% of materials with symptoms presented black discoloration of xylem vessels. This symptom was observed in materials from all stages of the propagation process analysed. Sectorial wood necrosis was the other symptom observed, also present in all stages, but with a markedly less incidence (around the 20% of materials with this symptoms). Additionally, grafted rooted plants (stage 4) also presented brown to black discolouration and necrosis from the base and necrotic lesions in the roots (**Figure 1**).

**Figure 2:**
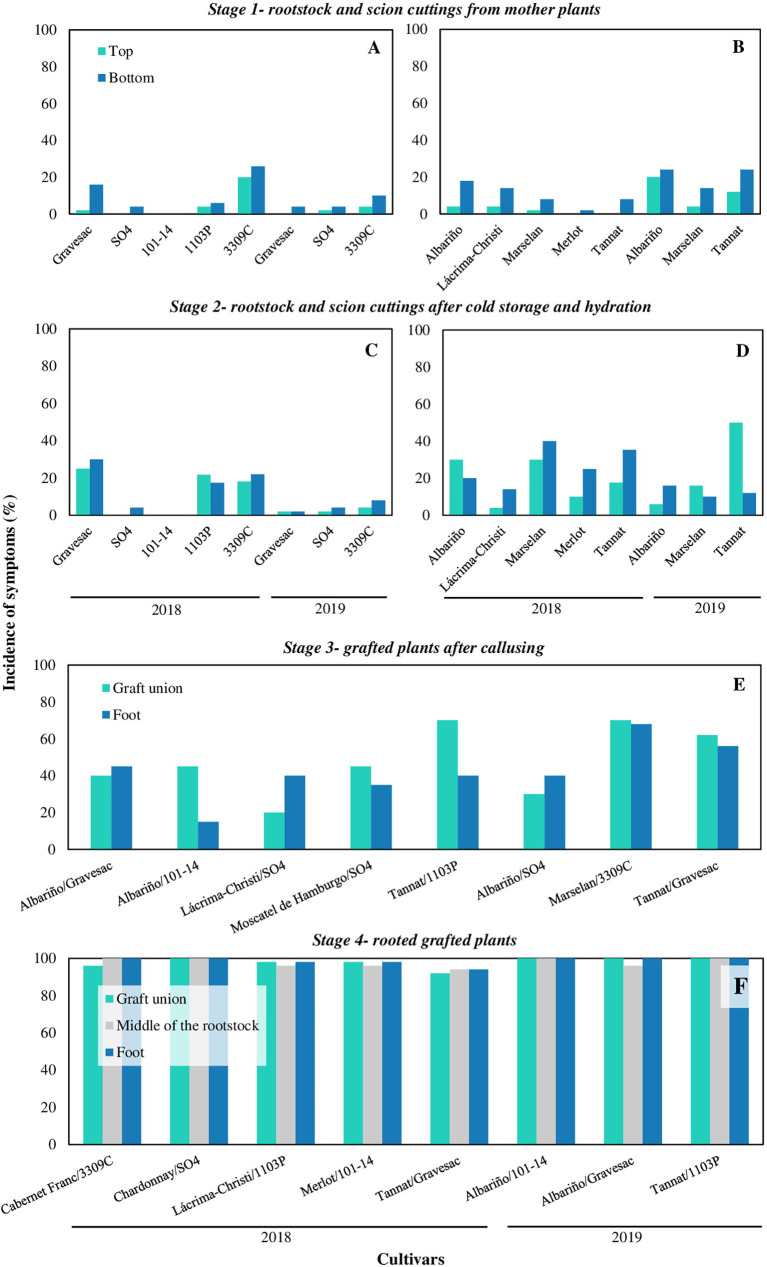
Incidence of grapevine trunk diseases symptoms in propagation materials of different cultivars, rootstocks, and combination cultivar-rootstocks throughout four stages of the nursery process in 2018 and 2019: **(A)** rootstocks and **(B)** scion cuttings from mother plants (stage 1); **(C)** rootstocks and **(D)** scion cuttings after cold storage and hydration (stage 2); **(E)** grafted plants after callusing (stage 3); **(F)** rooted grafted plants ready to plant (stage 4).

The incidence of symptoms increased throughout the nursery propagation process with a markedly increment in stage 4 (grafted rooted plants) (**Figure 2**). Considering all cultivars, in 2018 rootstock cuttings from stage 1 presented in average an incidence of symptoms of 5% at the top and 10% at the bottom, whereas the incidence on scion cuttings was 2% at the top and 10% at the bottom. In stage 2, rootstocks cuttings presented an average incidence of 13% and 15%, whereas scion cuttings showed 18% and 27% of incidence, at the top and at the bottom of the cuttings, respectively. Grafted plants in stage 3 had on average 44% of incidence in the grafted union and 35% in the basal part of the plant. Finally, plants in stage 4 showed on average 97%, 97% and 98% of incidence in the grafted union, middle part of the rootstock and in the foot, respectively. In 2019, the average incidence of symptoms in rootstock cuttings in stage 1 was 2% and 6%, whereas in scion cuttings was 12% and 21%, at the top and the bottom, respectively. Rootstock cuttings in stage 2 showed an incidence of 3% and 5%, whereas scion cuttings showed an incidence of 24% and 13%, at the top and at the bottom of the cuttings, respectively. Materials in stage 3 showed an average incidence of internal symptoms in the grafted union of 54% and 55% in the foot. Finally, plants in stage 4 had an average incidence of almost 100%, in all the three plant portions analysed (**Figure 2**).

### Fungal isolation and morphological identification

A total of 495 fungal isolates were obtained from grapevine, of which 236 were classified associated with BD, 181 to PD, 52 to BFD and 26 to DD.

Fungal isolates associated with BD were obtained from brown to dark streaks and sectorial wood necrosis. These isolates presented fast-growing, cottony aerial mycelium with white colour at the beginning turning to grey, dark grey or olive-green few days later. Approximately 80% of these isolates produced hyaline, aseptate and fusiform conidia resembling those produced by genera such as *Botryosphaeria* and *Neofusicoccum*, whereas the remaining 20% of isolates produced dark, aseptate and oblong to rounded apex conidia, which characterize *Diplodia* genus (Delgado et al., 2016). Fungal isolates of PD pathogens were obtained from brown to dark streaks and characterized by slow mycelial growth. The first sub-group formed by 100 *Ph. chlamydospora*-like isolates, presented sparse aerial mycelium, with colours ranging from green-olivaceous to olivaceous-black, abundant straight and pigmented conidia and dark green-brown conidiophores, characteristic of *Ph. chlamydospora* (Crous and Gams, 2000). The other sub-group was formed by 81 *Phaeoacremonium* spp. isolates, which showed a flat, pale to medium brown mycelium, abundant sporulation of hyaline and aseptate conidia and different types, sizes, and shapes of phialides, morphological characteristics of this genus (Mostert et al., 2006b). Isolates associated with BFD were obtained from brown to dark streaks and necrosis from the foot and from roots. These strains showed aerial and cottony mycelia ranging in colours from white to dark-yellow or slightly brown and produced macroconidia, microconidia and chlamydospores, resembling *Cylindrocarpon*-like asexual morphs (Chaverri et al., 2011; Agustí-Brisach and Armengol, 2013). Finally, isolates associated with DD were isolated predominantly from brown to dark streaks and characterized by moderate aerial mycelium white at first becoming light cream later and usually forming concentric rings with visible conidiomata at maturity containing alfa and beta conidia, morphologically resembling members of *Diaporthe* genus (Úrbez-Torres et al., 2013).

Considering both years of sampling, 18.2% of the total fungal isolates were obtained from asymptomatic wood tissues (90 strains). The 80% of these were associated with BD, 10% to PD and the remaining 10% to DD, whereas no isolates associated with BFD were obtained from asymptomatic tissues. Fungal isolates from asymptomatic tissues associated with BD and DD diseases accounted for 31% and 35% of total BD and DD isolates, respectively, whereas isolates associated with PD from asymptomatic tissues accounted for 5% of total PD isolates. Regarding the propagation stages from which they were isolated, 84.3% and 100% were obtained from materials at the first three stages (stages 1, 2 and 3) in 2018 and 2019, respectively.

### Molecular identification

A representative subsample of 180 strains isolated from symptomatic and asymptomatic materials was selected for molecular identification, attempting to include as much morphological diversity as possible within each group of fungi (**Supplementary Table 3**). BLAST comparisons grouped the isolates into eight fungal genera: *Botryosphaeria* (n=16), *Neofusicoccum* (n=21) and *Diplodia* (n=9) associated with BD, *Phaeoacremonium* (n=43) and *Phaeomoniella* (n=23) associated with PD, *Dactylonectria* (n=36) and *Ilyonectria* (n=6) associated with BFD, and *Diaporthe* (n=26) associated with DD.

The *Botryosphaeriaceae* phylogenetic analyses based on TEF sequences allowed the identification of seven species: *B. dothidea* (n=16), *N. parvum* (n=13), *D. pseudoseriata* (n=7), *N. cryptoaustrale* (n=3), *N. luteum* (n=3), *D. seriata* (n=2) and *N. australe* (n=1) (**Supplementary Figures 1-3**). The *Dactylonectria* and *Ilyonectria* phylogenetic analyses using HIS3 sequences, enabled us to identify seven species including *Da. novozelandica* (n=15), *Da. macrodidyma* (n=11), *Da. torresensis* (n=6), *Da. pauciseptata* (n=3), *Da. valentina* (n=1), *I. liriodendri* (n=4), *I. robusta* (n=1), whereas one strain was identified as *Ilyonectria* sp. (n=1) (**Supplementary Figures 4** and **5**).

The *Phaeoacremonium* phylogenetic analysis based on TUB2 sequences allowed us to identify three species, *P. minimum* (n=41), *P. austroafricanum* (n=1) and *P. oleae* (n=1). Regarding *Phaeomoniella chlamydospora*-like isolates, phylogenetic analysis based on ITS sequences grouped all our isolates (n=23) with *Ph. chlamyd*ospora ex-type strain (**Supplementary Figures 6** and **7**).

Finally, the *Diaporthe* phylogenetic analysis, based on TEF sequences, allowed the identification of *Di. baccae* (n=2), *Di. eres* (n=2), *Di. foeniculina* (n=2) and *Di. terebinthifolii* (n=1), while the remaining 19 strains of this group, could not be distinguished by this single locus. These 19 strains fell within a *Di. ampelina*/*hungariae*/*hispaniae* clade with high bootstrap support (1 Bayesian posterior probability and 97% Maximum likelihood) (**Supplementary Figure 8**).

### Fungal Trunk Diseases Incidence

In both sampling years, the incidence of GTDs increased noticeably as the grapevine propagation process progressed. BD pathogens were found in materials from the four stages analysed. The total incidence of BD in 2018 was 2.9% and 6.4% in rootstock and scion cuttings, respectively, from stage 1, whereas in 2019 it was 15% in rootstock and 27.4% in scion cuttings (**Table 2**). In materials from stage 2, the total incidence in rootstock was 2.7% and 8.3%, in 2018 and 2019, respectively, and 15% in scion cuttings from both sampling years. In propagation materials from stage 3, the total incidence of BD was 41% in 2018 and 28% in 2019, being the incidence remarkably higher at the graft union compared with the base of the plant. In stage 4, BD showed an incidence of 55.1% in 2018 and 36.7% in 2019, being these pathogens isolated from all plant portions tested (**Table 3**).

**Table 2:** Incidence of fungal grapevine trunk diseases in rootstock and scion cuttings from mother plants (stage 1) and rootstock and scion cuttings after cold storage and hydration (stage 2).

		Disease incidence % (number of infected samples/total samples)											
		Stage 1						Stage 2					
		Rootstock			Scion			Rootstock			Scion		
Year	Fungal disease^1^	Top^2^	Bottom	Total	Top	Bottom	Total	Top	Bottom	Total	Top	Bottom	Total
2018	PD	1.9 (2/104)	1.0 (1/104)	2.9 (3/104)	–	0.8 (1/125)	0.8 (1/125)	–	–	–	–	–	–
	BFD	–	–	–	–	–	–	–	–	–	–	–	–
	BD	1.0 (1/104)	1.9 (2/104)	2.9 (3/104)	1.6 (2/125)	4.8 (6/125)	6.4 (8/125)	1.8 (2/111)	0.9 (1/111)	2.7 (3/111)	7.0 (7/100)	8.0 (8/100)	15.0 (15/100)
	DD	–	1.0 (1/104)	1.0 (1/104)	–	0.8 (1/125)	0.8 (1/125)	0.9 (1/111)	–	0.9 (1/111)	1.0 (1/100)	4.0 (4/100)	5.0 (5/100)
2019	PD	–	–	–	1.4 (1/73)	1.4 (1/73)	2.7 (2/73)	–	–	–	–	–	–
	BFD	–	–	–	–	–	–	–	–	–	–	–	–
	BD	6.7 (4/60)	8.3 (5/60)	15.0 (9/60)	8.2 (6/73)	19.2 (14/73)	27.4 (20/73)	5.0 (3/60)	3.3 (2/60)	8.3 (5/60)	11.7 (7/60)	3.3 (2/60)	15.0 (9/60)
	DD	–	–	–	1.4 (1/73)	5.5 (4/73)	6.8 (5/73)	–	–	–	–	–	–

^1^PD, Petri disease; BFD, Black-foot disease; BD, Botryosphaeria dieback; DD, Diaporthe dieback.

^2^The “top” margin of rootstock and scion cuttings was the part of the 1.1m long cutting furthest from the mother trunk (at 0.95-1.1 m from the mother trunk), whereas the “bottom” margin was the part of the cutting nearest to the mother trunk (the first 0.15 m of the cutting).

**Table 3:** Incidence of fungal grapevine trunk diseases in grafted plants after callusing (stage 3) and in rooted grafted plants (stage 4).

			Disease incidence % (number infected of samples/total samples)						
			Stage 3			Stage 4			
Year	Fungal disease^1^		Graft union^2^	Foot	Total	Graft union	Middle part of the rootstock	Foot and roots^3^	Total
2018	PD	1.0 (1/100)		–	1.0 (1/100)	45.9 (45/98)	18.4 (18/98)	38.8 (38/98)	56.1 (55/98)
	BFD	–		–	–	–	–	14.3 (14/98)	14.3 (14/98)
	BD	38.0 (38/100)		4.0 (4/100)	41.0 (41/100)	24.5 (24/98)	39.8 (39/98)	8.2 (8/98)	55.1 (54/98)
	DD	2.0 (2/100)		–	2.0 (2/100)	3.1 (3/98)	4.1 (4/98)	1.0 (1/98)	8.2 (8/98)
2019	PD	2.7 (2/75)		4.0 (3/75)	6.7 (5/75)	40.0 (24/60)	31.7 (19/60)	40.0 (24/60)	58.3 (35/60)
	BFD	–		–	–	3.3 (2/60)	–	60.0 (36/60)	60.0 (36/60)
	BD	25.3 (19/75)		4.0 (3/75)	28.0 (21/75)	21.7 (13/60)	15.0 (9/60)	11.7 (7/60)	36.7 (22/60)
	DD	1.3 (1/75)		–	1.3 (1/75)	1.7 (1/60)	1.7 (1/60)	–	3.3 (2/60)

^1^ PD, Petri disease; BFD, Black-foot disease; BD, Botryosphaeria dieback; DD, Diaporthe dieback.

^2^ The “Graft union” section includes the portion above the graft union (scion) and the “Foot” is the basal part of the plant. In stage 4, the “Middle part of the rootstock” refers to the portion of the plant between the graft union and the foot.

^3^ Only isolates associated with BFD and PD were obtained from roots.

Regarding PD, in 2018 rootstock cuttings from stage 1 showed a total incidence of 2.9%, whereas scion cuttings presented a total incidence of 0.8%. In 2019, scion cuttings from stage 1 showed a total PD incidence of 2.7%, whereas no isolates associated with PD were obtained from rootstock cuttings (**Table 2**). Also, no PD isolates were obtained from analysed materials at the stage 2 in neither of the two years of evaluation. At the stage 3, the total incidence was 1% in 2018 and 6.7% in 2019, whereas in plants from stage 4, the incidence of PD was 56.1% and 58.3%, in 2018 and 2019, respectively, considering all the portions of the plant analysed. Pathogens associated with this disease were isolated from all plant portions tested in stage 4 (**Table 3**).

The total incidence of DD in 2018 was 1.0% and 0.8% in rootstock and scion cuttings from stage 1, respectively, whereas in stage 2, the incidence was 0.9% and 5.0%, in rootstock and scion cuttings, respectively. In 2019, isolates of *Diaporthe* from stage 1 were only obtained from scion cuttings, with a total incidence of 6.8%, while no isolates were obtained from materials in stage 2 (**Table 2**). In propagation materials from stage 3, DD showed an incidence of 2% in 2018 and 1.3% in 2019, whereas in stage 4 the incidence was 8.2% in 2018 and 3.3% in 2019 (**Table 3**).

Regarding BFD, these pathogens were isolated only from stage 4. The incidence was 14.3% in 2018 and 60% in 2019 and were isolated from the foot and roots of the plants, except from two strains that were isolated from the graft union in 2019 (**Table 3**). From roots, only pathogens associated with BFD and PD were isolated.

Infected materials in stages 1, 2 and 3 had only one disease. However, in grafted rooted plants in stage 4, combinations of up to four diseases were found to affect the same plant simultaneously in 2019. From the results of isolations, the total incidence of GTDs in materials from stage 4 was 84.7% in 2018 and 83.3% in 2019. The most prevalent GTD combination was BD with PD in 2018 (26.5% of the plants) and PD with BFD in 2019 (18.3% of the plants) (**Table 4**).

**Table 4:** Incidence of grapevine trunk diseases on grafted rooted vines ready to plant (stage 4) based on fungal isolations .

	Frequency of infected plants(% disease incidence)	
Fungal disease	2018	2019
Alone		
Petri disease (PD)	21.4 (21/98)	16.7 (10/60)
Black-foot disease (BFD)	4.1 (4/98)	10.0 (6/60)
Botryosphaeria dieback (BD)	15.3 (15/98)	1.7 (1/60)
Diaporthe dieback (DD)	–	–
In combination		
PD + BFD	3.1 (3/98)	18.3 (11/60)
PD + BD	26.5 (26/98)	5.0 (3/60)
PD + DD	–	–
BD + BFD	5.1 (5/98)	13.3 (8/60)
BD + DD	3.1 (3/98)	–
PD + BFD + BD	1.0 (1/98)	15.0 (9/60)
PD + BD + DD	4.1 (4/98)	–
PD + BFD + DD	1.0 (1/98)	1.7 (1/60)
PD + BFD + BD + DD	–	1.7 (1/60)
Total	84.7 (83/98)	83.3 (50/60)

## Discussion

In this study, we have examined the incidence of GTDs in grapevine planting material, throughout the four stages of the local nursery propagation process, based on the presence of internal wood symptoms and GTD pathogens. We have been particularly interested in understanding the health status of the vines produced, which GTD pathogens affect the planting material and the steps of the local nursery process in which vines are infected. To the best of our knowledge, this is the first comprehensive study in South America showing the role of infected planting material in the dissemination of GTDs.

Our results show that there is a high incidence of GTDs in nursery grapevines produced in Uruguay, regardless of the scion/rootstock combination. The 84.7% and 83.3% of the examined finished nursery vines were affected by at least one GTD-associated fungus, in 2018 and 2019, respectively. These results highlight the role of infected locally produced vines as one of the main ways of dissemination of GTD pathogens. Several studies have also confirmed GTDs affecting nursery vines with varying incidence in Spain (Aroca et al., 2006; Giménez-Jaime et al., 2006; Agustí-Brisach et al., 2013a; Pintos et al., 2018; Berlanas et al., 2020; Maldonado-González et al., 2020), Portugal (Rego et al., 2009), Italy (Spagnolo et al., 2011; Carlucci et al., 2017), South Africa (Halleen et al., 2003; Halleen et al., 2006) and France (Spagnolo et al., 2011). Furthermore, noticeable incidence of GTDs in nursery grapevines was also observed by Pintos et al. (2018) in Spain, who found that the incidence of at least one GTD-associated fungus affecting grafted rooted vines was 93%.

Incidence of symptoms and GTD-associated fungi increased throughout the four stages evaluated, suggesting that fungal infections occurred during the propagation process, which is consistent with previous studies (Gramaje and Armengol, 2011; Gramaje et al., 2022). Firstly, typical internal wood symptoms of GTDs were observed and pathogens associated with PD, BD and DD diseases were isolated in low frequency from scion and rootstocks cuttings from mother plants, as it has been described in other grapevine world regions (Pascoe and Cottral, 2000; Rego et al., 2001; Ridgway et al., 2002; Edwards and Pascoe, 2004; Fourie and Halleen, 2004; Whiteman et al., 2007; Zanzotto et al., 2007; Aroca et al., 2010; Billones-Baaijens et al., 2013). Therefore, our results also provided evidence that scion and rootstocks cuttings used for propagation are a primary source of GTD pathogens. Then, after cold storage and hydration for 24-48 hours in water, the scion and rootstocks cuttings generally showed a slight increase in the incidence of GTDs internal symptoms. Several studies have detected the presence of PD and BD pathogens in the water of hydration tanks using both, molecular detection, and culture dependent approaches, suggesting that during soaking step infections can occur (Retief et al., 2006; Edwards et al., 2007; Pollastro et al., 2009; Vigues et al., 2009; Aroca et al., 2010; Gramaje et al., 2011). Moreover, Waite et al. (2013) confirmed that soaking cuttings is a potential source of cross contamination of field-acquired microorganisms and found that this can occur after relatively short periods of soaking. Therefore, it is likely that some infections occurred during the hydration stage, but they were not visible at the time of sampling due to the slow development of GTDs symptoms in the host.

Grafting and callusing stages have been widely identified as key steps of the propagation process in which new infections occur (Gramaje and Armengol, 2011). Our results also showed an increment of GTDs symptoms and isolated pathogens after grafting and callusing, suggesting that new infections may have occurred at this stage. These steps involve many cuts and wounds, as a consequence of the disbudding, grafting and improperly matched graft unions, which make the material susceptible to be infected by GTD-associated fungi (Giménez-Jaime et al., 2006; Gramaje and Armengol, 2011). In addition, several investigations detected the presence of PD pathogens in washings of scissors used to cut buds and grafting machines (Whiteman et al., 2004; Retief et al., 2006; Pollastro et al., 2009; Aroca et al., 2010; Gramaje et al., 2011), indicating that pathogen dissemination also occurs during this stage. Moreover, the environmental conditions of high temperature and humidity in callusing rooms, are favorable for the growth and wood colonization of pathogens (Gramaje and Armengol, 2011).

The main GTDs found affecting nursery vines ready to plant were PD, BFD and BD, alone or in combination. The prevalence of PD and BFD was expected, as a big amount of research indicated that these are the main GTDs affecting nursery and young vines in the world (Rego et al., 2000; Halleen et al., 2003; Gramaje and Armengol, 2011; Gramaje et al., 2018), and in Uruguay (Abreo et al., 2010; Abreo et al., 2011). As well as this, the high incidence of BD was unsurprising, as *Botryosphaeriaceae* species were recently the most frequently detected GTD-associated fungi affecting nursery vines in Spain (Pintos et al., 2018) and Italy (Carlucci et al., 2017). The simultaneous presence of these diseases in vines ready to plant is especially worrisome because any stress factor may trigger more rapidly the symptoms of decline in the vineyard (Hrycan et al., 2020).

Black-foot pathogens were exclusively isolated from vines after the stage of rooting in the nursery field. This result agrees with several studies that have indicated that BFD pathogens rarely occur in the propagation material prior to the rooting stage in nursery fields (Rego et al., 2001; Fourie and Halleen, 2002; Halleen et al., 2003; Fourie and Halleen, 2004; Halleen et al., 2006; Halleen et al., 2007; Agustí-Brisach et al., 2013a). Based on our culture-dependent approach, results make emphasis on the fact that the nursery field is the primary source of inoculum of BFD, as it was also previously demonstrated (Rego et al., 2001; Halleen et al., 2006; Agustí-Brisach et al., 2013b; Agustí-Brisach et al., 2014; Berlanas et al., 2017). However, some research based on molecular techniques also detected BFD inoculum in the water of hydration tanks, washings of cutting and grafting tools, callusing media, and grafted plants after callusing, suggesting that infections of BFD-associated fungi can also occur during these earlier stages or at least may spread (Agustí-Brisach et al., 2013a; Cardoso et al., 2013). On the other hand, a marked increase in PD incidence was also observed in rooted grafted vines, compared with materials from earlier stages. Several researchers have been reported the presence of PD inoculum in nursery and vineyards soil (Rooney et al., 2001; Mostert et al., 2006a; Retief et al., 2006; Agustí-Brisach et al., 2013b; Maldonado-González et al., 2020), which, together with the wounds generated during the rooting process and the probable incomplete callusing of the basal end of the rootstock (Gramaje and Armengol, 2011), could explain the registered increment.

The increment of internal symptoms and GTD-associated fungi isolated after the rooting stage could be mainly explained by the occurrence of new infections throughout the growing season in the nursery field. However, it is also necessary to emphasize that GTD pathogens can appear as endophytes or latent pathogens and grow over a larger area within the nursery stock as the material ages before symptoms develop (Halleen et al., 2003; Aroca et al., 2010; Abreo et al., 2013; Úrbez-Torres et al., 2015; Berlanas et al., 2020; Hrycan et al., 2020). In fact, although culture-dependent approaches require a high level of colonization of the pathogen in the host to be detected by isolation (Agustí-Brisach et al., 2013a), we also isolated GTD pathogens from asymptomatic material earlier in the propagation process, mainly BD pathogens, confirming that these pathogens can occur as latent or endophytes in nursery material. Therefore, in stage 4 we observed an accumulation of infections that occurred during the propagation process, including those that were not previously visible.

Based on the phylogenetic analysis of a single locus, the 180 isolates selected were placed into eight genera and 89% identified within 22 fungal species associated with GTDs in the nursery grapevine material. Within the *Botryosphaeriaceae* group we found *N. cryptoaustrale* and *D. pseudoseriata* as new records on grapevine in Uruguay. Regarding BFD pathogens, *Da. novozelandica*, *Da. torresensis*, *Da. valentina* and *I. robusta* are new records on grapevine in this country. It should be noted that the etiology of BFD has been subjected to taxonomic revision in recent years (Chaverri et al., 2011; Cabral et al., 2012a; Cabral et al., 2012b; Lombard et al., 2014). Thus, the main causal agents of BFD identified previously as *Cylindrocarpon*-like asexual morphs, are now identified as belonging to the genera *Ilyonectria* and *Dactylonectria*. Therefore, the additional fungal species detected in this work, *Da. macrodidyma*, *Da. pauciseptata* and *I. liriodendri*, do not represent new records as they were previously reported associated with BFD in Uruguay like *Cylindrocarpon macrodidymum*, *C. pauciseptatum* and *C. liriodendri* by Abreo et al. (2010). Regarding PD pathogens, *P. austroafricanum* is a new record on grapevine in Uruguay and, to the best of our knowledge, this is the first report of *P. oleae* associated with PD on grapevine worldwide. *Phaeoacremonium oleae* was isolated from a symptomatic grafted rooted plant. The species was described by Spies et al. (2018) and has been reported associated with olive trunk diseases in South Africa (Spies et al., 2018) and Italy (Raimondo et al., 2021). Finally, within the *Diaporthe* genus, *Di. baccae*, *Di. eres*, *Di. foeniculina* are new records on grapevine in Uruguay, and as far as we know, this is the first record of *Di. terebinthifolii* on grapevine worldwide. *Diaporthe terebinthifolii* was described by Gomes et al. (2013) and was isolated for first time as an endophytic specie from the leaf of *Schinus terebinthifolius* in Brazil. In this work, it was isolated from an asymptomatic scion cutting, thus, it would be necessary to test the pathogenicity on grapevine. For *Diaporthe* strains that could not be identified based on the phylogeny of the single TEF locus, it will be essential to develop a multi-locus phylogeny analysis for their identification. In recent works, three and up to seven loci have been employed to accurately identified *Diaporthe* species (Guarnaccia et al., 2018; Manawasinghe et al., 2019; León et al., 2020; Dong et al., 2021; Jimenez et al., 2022).

In conclusion, our results showed that the current health status of nursery vines produced in Uruguay may endanger the longevity of the vineyard from the start. Further research is needed to assess how many of these infected nursery vines develop foliar symptoms in the vineyard. Nevertheless, our results suggested that a sanitation programme is required to reduce the incidence of GTDs on nursery vines. It has been proposed that an integrated and holistic management program, including biological, physical, chemical, and other strategies, is the most effective way to reduce GTD pathogens infections in the nursery (Halleen and Fourie, 2016; Gramaje et al., 2018). Currently, GTD pathogens are not included in the grapevine nursery certification program of Uruguay (INASE, 2006). The establishment of tolerance limits for the presence of fungal trunk pathogens in nursery vines could help to attempt to control these diseases.

## Data Availability Statement

The datasets presented in this study can be found in online repositories. The names of the repository/repositories and accession number(s) can be found in the article/**Supplementary Material**.

## Author Contributions

SA conceived, designed, and directed the investigation. MC performed the experimental work, the analyses of data and wrote the draft of the manuscript. SA and PM supervised the experimental work, the data analyses and made a critical review of the manuscript. MG contributed to the experimental work. VM contributed to the data analyses and made a critical review of the manuscript. All authors contributed to and approved the final manuscript.

## Funding

The research was funded by CSIC (Comisión Sectorial de Investigación Científica), under the project “Incidence, etiology and origin of wood diseases during the production process of grapevine plants in the main nursery of Uruguay”. María Julia Carbone is a doctorate student of PEDECIBA-Biology (Programa de Desarrollo de las Ciencias Básicas) and was supported by grants of ANII (Agencia Nacional de Investigación e Innovación, grant agreement No POS_NAC_2017_1_141446) and CAP (Comisión Académica de Posgrado).

## Acknowledgments

We thank the nursery owner and employees for facilitating and providing the grapevine materials.

## Conflict of Interest

The authors declare that the research was conducted in the absence of any commercial or financial relationships that could be construed as a potential conflict of interest.

## Publisher’s Note

All claims expressed in this article are solely those of the authors and do not necessarily represent those of their affiliated organizations, or those of the publisher, the editors and the reviewers. Any product that may be evaluated in this article, or claim that may be made by its manufacturer, is not guaranteed or endorsed by the publisher.
