# Supplementary material for: Grapevine nursery propagation material as source of fungal trunk disease pathogens in Uruguay
Source: Front Fungal Biol. 2022 Jul 22;3:958466. doi: 10.3389/ffunb.2022.958466 (PMC10512308; doi:10.3389/ffunb.2022.958466)
Supplement: Supplementary file 1 [file DataSheet_1.docx]

Supplementary Material

# Supplementary Figures


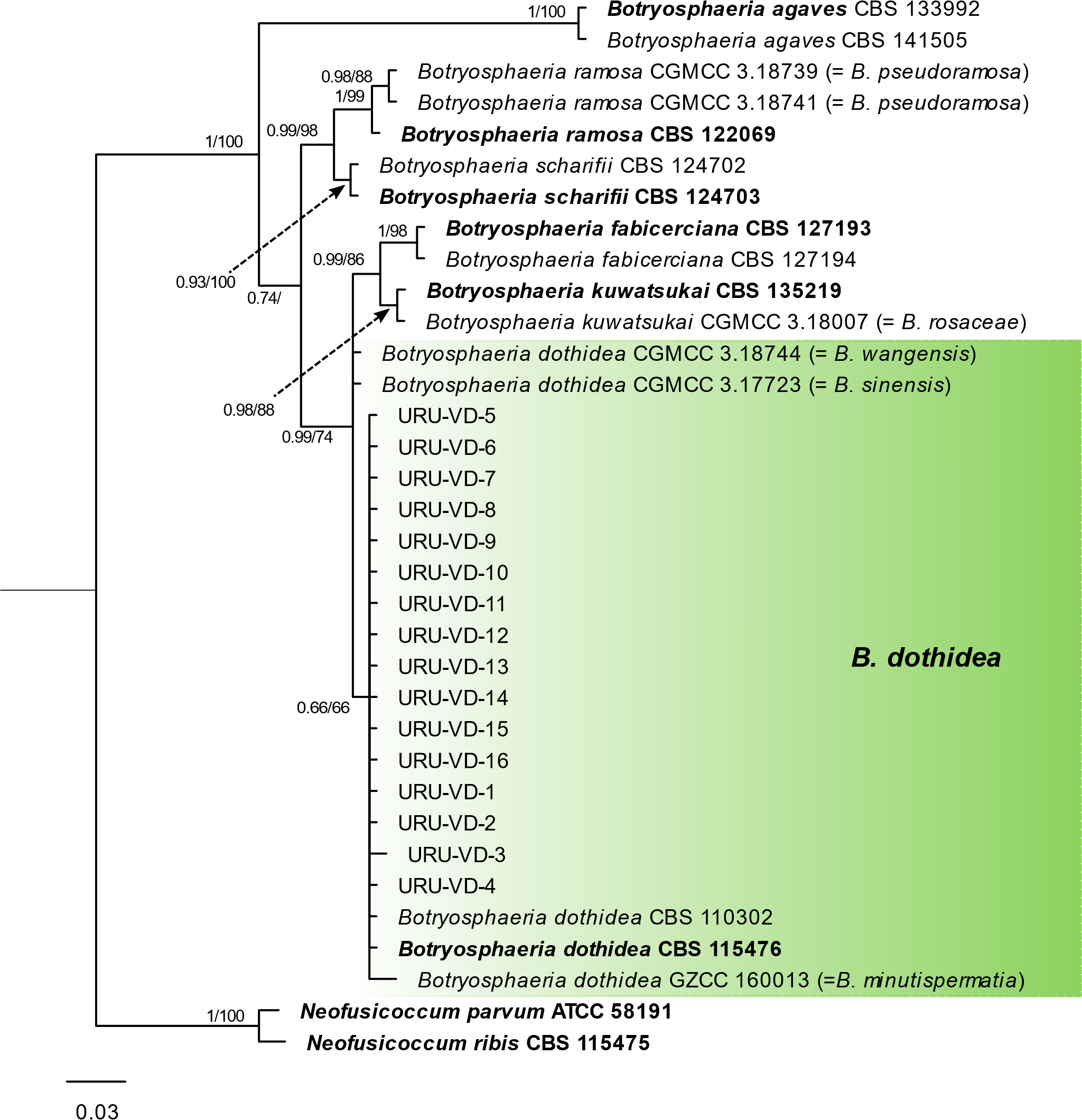


**Supplementary Figure 1.** Bayesian inference phylogenetic tree inferred from the elongation factor region (TEF) of 16 *Botryosphaeria* strains isolated from grapevine propagation materials and sequences obtained from the GenBank (ex-type indicated in bold). *Neofusicoccum parvum* ATCC58191 and *Neofusicoccum ribis* CBS 115475 were used as outgroup. Bootstrap support values of posterior probability and maximum likelihood higher than 0.50 and 50 are shown at the nodes before and after the bar, respectively. The scripts indicate that the nodes do not exist in the maximum likelihood tree. Scale bar represents the estimated number of substitutions per site.


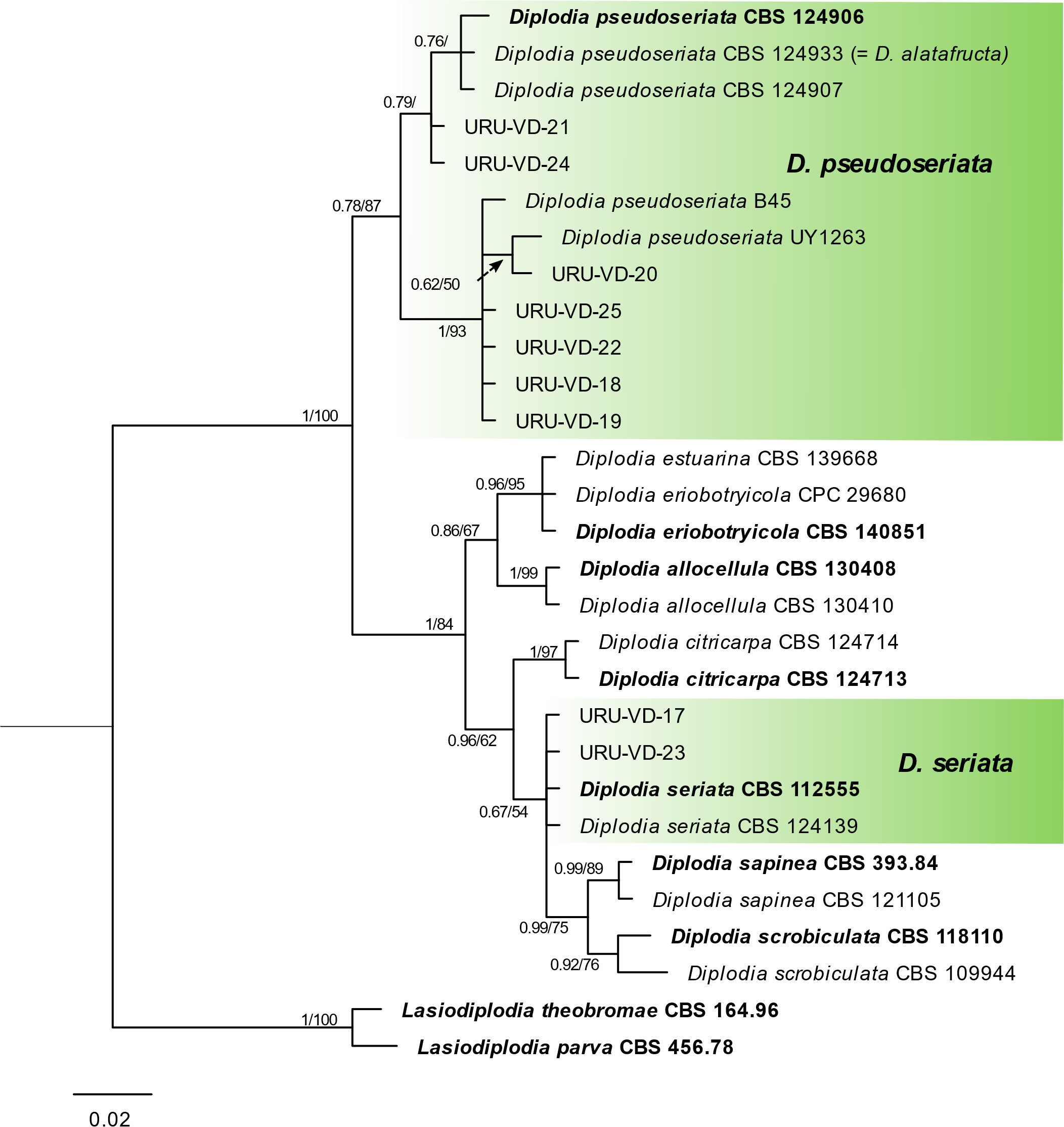


**Supplementary Figure 2.** Bayesian inference phylogenetic tree inferred from the elongation factor region (TEF) of 9 *Diplodia* strains isolated from grapevine propagation materials and sequences obtained from the GenBank (ex-type indicated in bold). *Lasiodiplodia theobromae* CBS 164.96 and *Lasiodiplodia parva* CBS 456.78 were used as outgroup. Bootstrap support values of posterior probability and maximum likelihood higher than 0.50 and 50 are shown at the nodes before and after the bar, respectively. The scripts indicate that the nodes do not exist in the maximum likelihood tree. Scale bar represents the estimated number of substitutions per site.


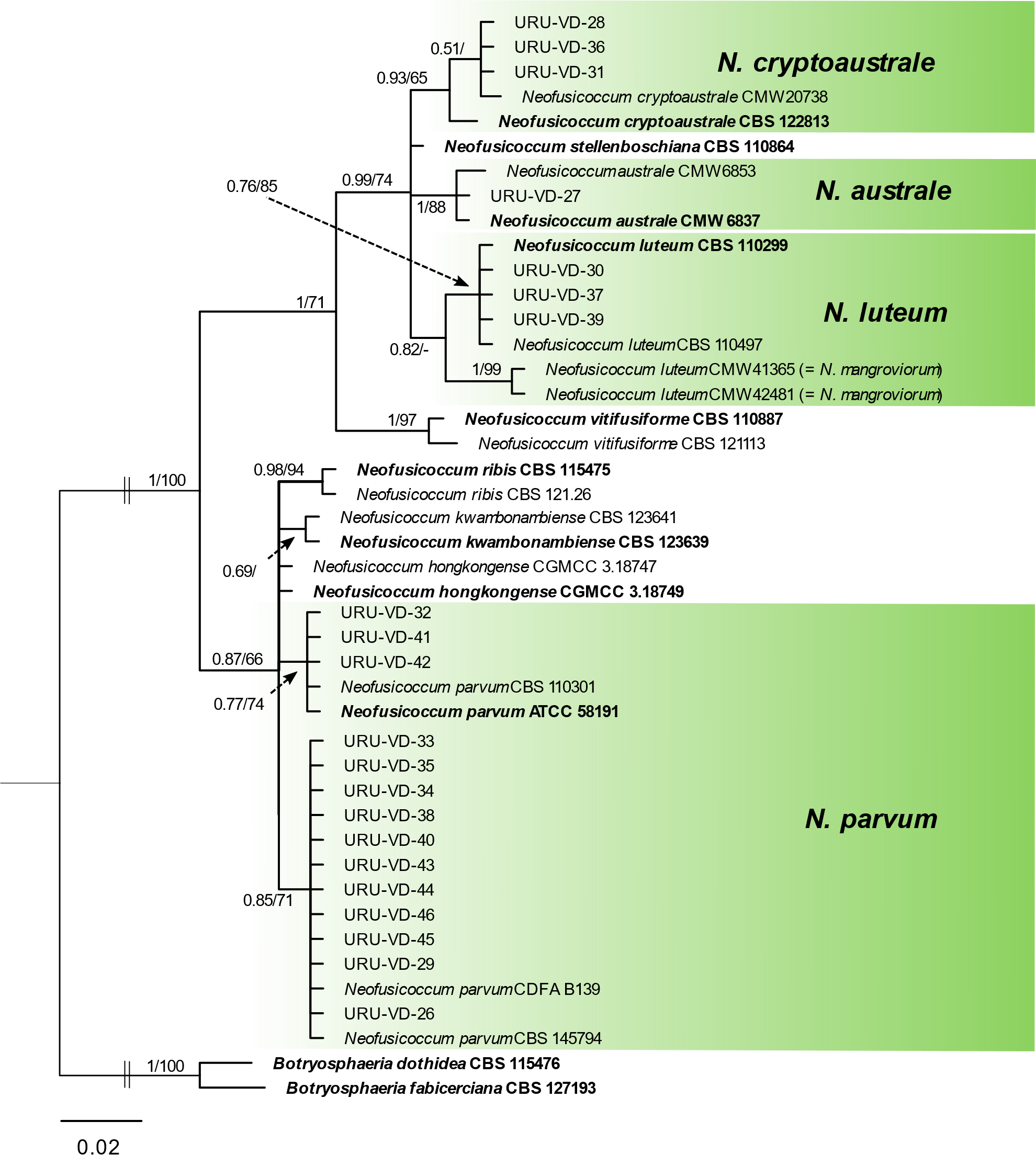


**Supplementary Figure 3.** Bayesian inference phylogenetic tree inferred from the elongation factor region (TEF) of 21 *Neofusicoccum* strains isolated from grapevine propagation materials and sequences obtained from the GenBank (ex-type indicated in bold). *Botryosphaeria dothidea* CBS 115476 and *Botryosphaeria fabicerciana* CBS 127193 were used as outgroup. Bootstrap support values of posterior probability and maximum likelihood higher than 0.50 and 50 are shown at the nodes before and after the bar, respectively. The scripts indicate that the nodes do not exist in the maximum likelihood tree. Double hash marks indicate branch lengths shortened at least 2-fold to facilitate visualization. Scale bar represents the estimated number of substitutions per site.


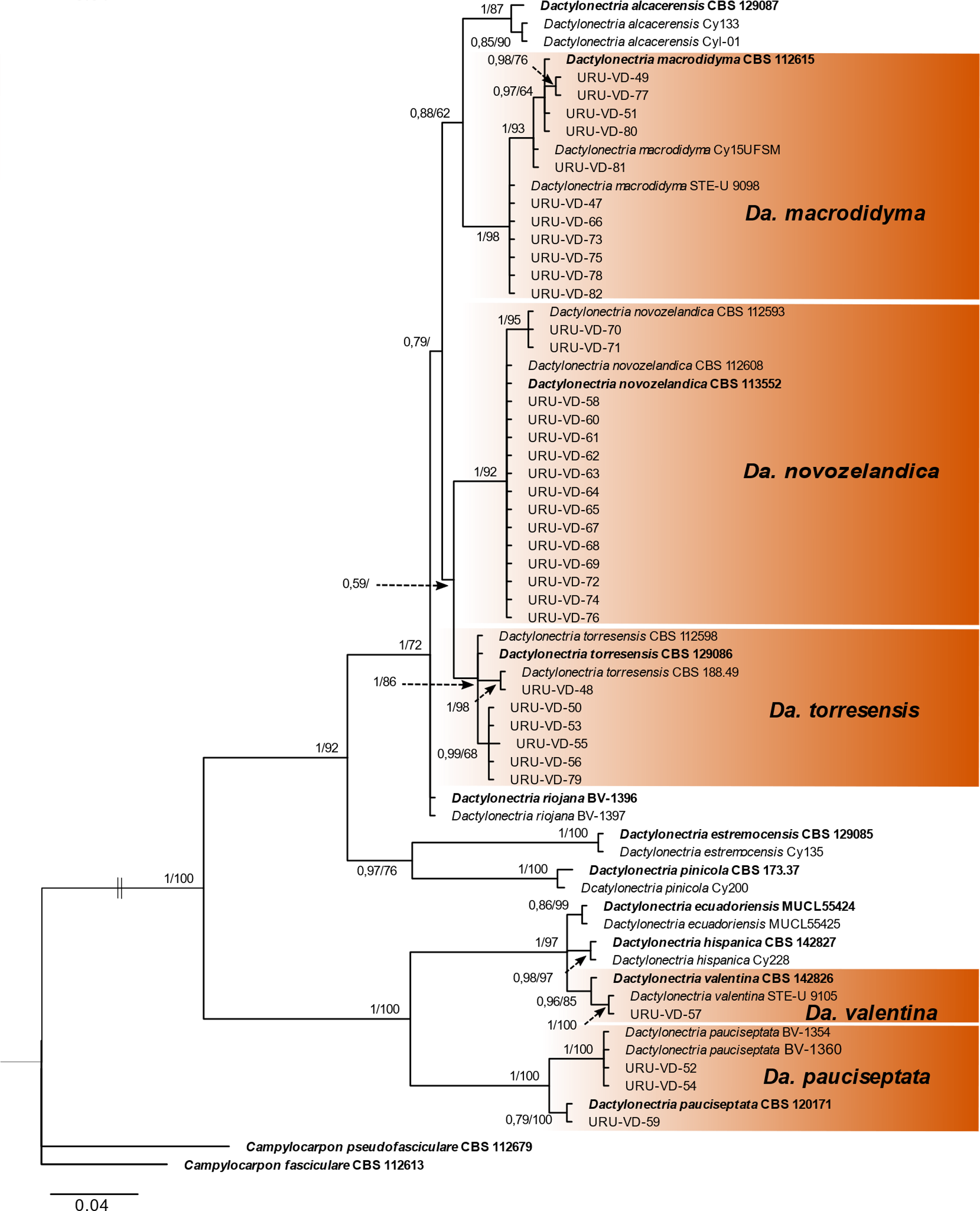


**Supplementary Figure 4.** Bayesian inference phylogenetic tree inferred from the histone 3 gene region (HIS3) of 36 *Dactylonectria* strains isolated from grapevine propagation materials and sequences obtained from the GenBank (ex-type indicated in bold). *Campylocarpon fasciculare* CBS 112613 and *Campylocarpon pseudofasciculare* CBS 112679 were used as outgroup. Bootstrap support values of posterior probability and maximum likelihood higher than 0.50 and 50 are shown at the nodes before and after the bar, respectively. The scripts indicate that the nodes do not exist in the maximum likelihood tree. Double hash marks indicate branch lengths shortened at least 2-fold to facilitate visualization. Scale bar represents the estimated number of substitutions per site.


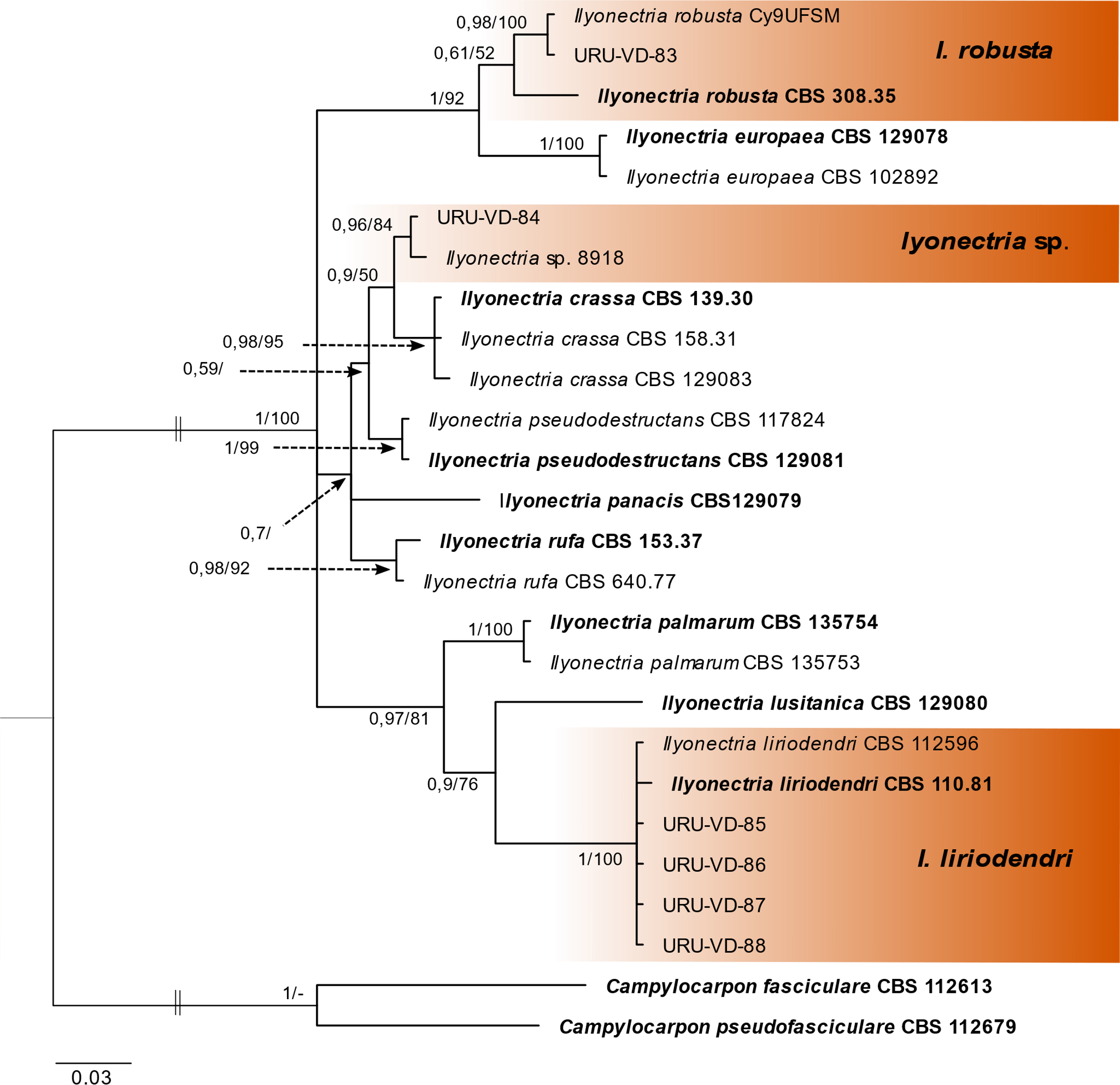


**Supplementary Figure 5.** Bayesian inference phylogenetic tree inferred from the histone 3 gene region (HIS3) of 6 *Ilyonectria* strains isolated from grapevine propagation materials and sequences obtained from the GenBank (ex-type indicated in bold). *Campylocarpon fasciculare* CBS 112613 and *Campylocarpon pseudofasciculare* CBS 112679 were used as outgroup. Bootstrap support values of posterior probability and maximum likelihood higher than 0.50 and 50 are shown at the nodes before and after the bar, respectively. The scripts indicate that the nodes do not exist in the maximum likelihood tree. Double hash marks indicate branch lengths shortened at least 2-fold to facilitate visualization. Scale bar represents the estimated number of substitutions per site.


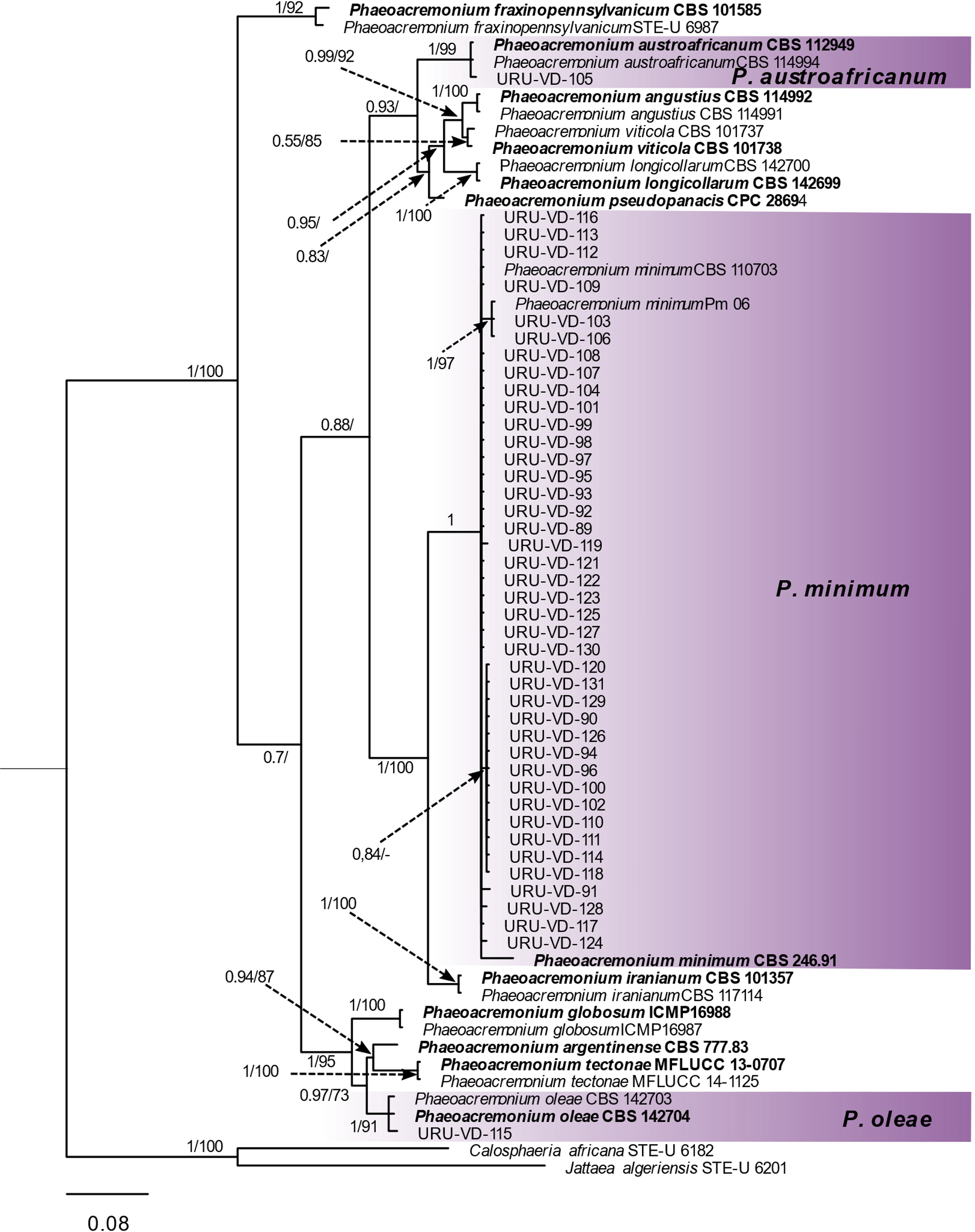


**Supplementary Figure 6.** Bayesian inference phylogenetic tree inferred from the beta-tubulin gene region (TUB2) of 43 *Phaeoacremonium* strains isolated from grapevine propagation materials and sequences obtained from the GenBank (ex-type indicated in bold). *Calosphaeria africana* STE-U 6182 and *Jattaea algeriensis* STE-U 6201 were used as outgroup. Bootstrap support values of posterior probability and maximum likelihood higher than 0.50 and 50 are shown at the nodes before and after the bar, respectively. The scripts indicate that the nodes do not exist in the maximum likelihood tree. Scale bar represents the estimated number of substitutions per site.


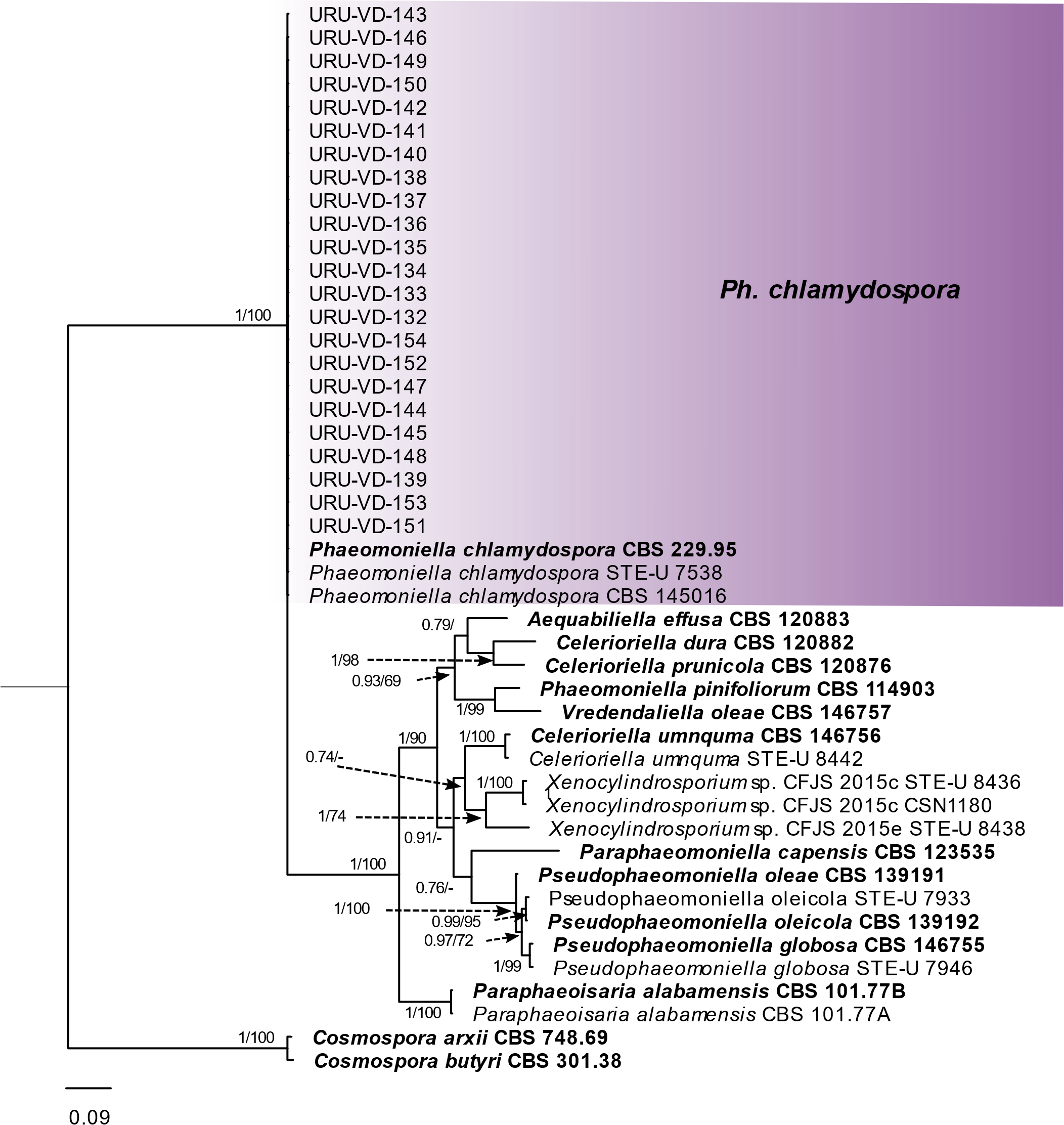


**Supplementary Figure 7.** Bayesian inference phylogenetic tree inferred from the internal transcribed spacer region and 5.8S rRNA (ITS) of 23 *Phaeomoniella chlamydospora* strains isolated from grapevine propagation materials and sequences obtained from the GenBank (ex-type indicated in bold). *Cosmospora arxii* CBS 748.69 and *Cosmospora butyri* CBS 301.38 were used as outgroup. Bootstrap support values of posterior probability and maximum likelihood higher than 0.50 and 50 are shown at the nodes before and after the bar, respectively. The scripts indicate that the nodes do not exist in the maximum likelihood tree. Scale bar represents the estimated number of substitutions per site.


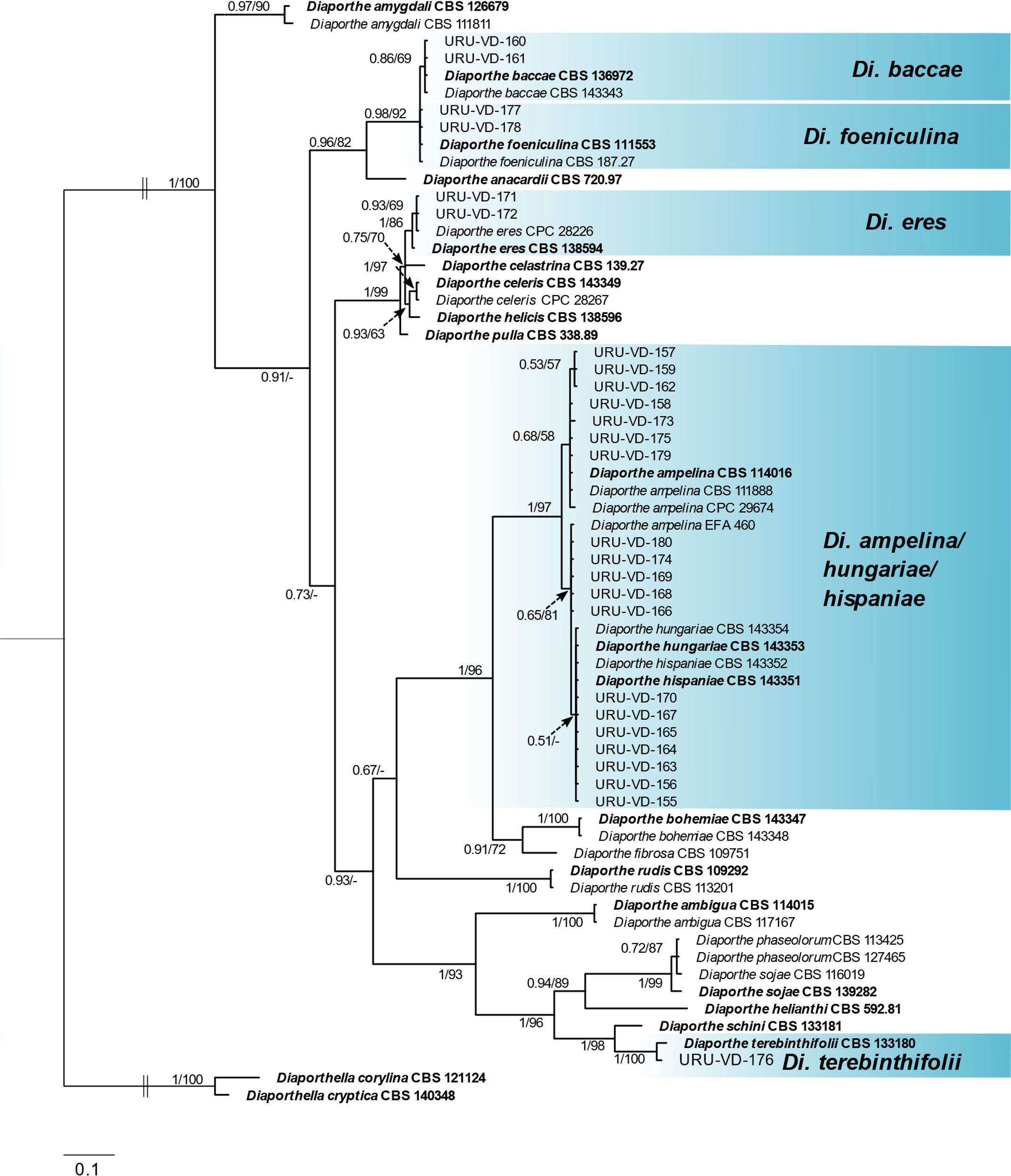


**Supplementary Figure 8.** Bayesian inference phylogenetic tree inferred from the elongation factor region (TEF) of 26 *Diaporthe* strains isolated from grapevine propagation materials and sequences obtained from the GenBank (ex-type indicated in bold). *Diaporthella corylina* CBS 121124 and *Diaporthella cryptica* CBS 140348 were used as outgroup. Bootstrap support values of posterior probability and maximum likelihood higher than 0.50 and 50 are shown at the nodes before and after the bar, respectively. The scripts indicate that the nodes do not exist in the maximum likelihood tree. Double hash marks indicate branch lengths shortened at least 2-fold to facilitate visualization. Scale bar represents the estimated number of substitutions per site.

# Supplementary Tables

# Supplementary Table 1. Accession numbers of DNA sequences obtained from the GenBank and used for the phylogenetic analyses

| **Taxon** | **Fungal species** | **Strain number** | **Host** | **GenBank accesion no.** | | | |
| --- | --- | --- | --- | --- | --- | --- | --- |
|  |  |  |  | **TEF** | **HIS3** | **TUB2** | **ITS** |
| ***Botryosphaeria*** | ***Botryosphaeria agaves*** | **CBS 133992** | *Agave* sp. | JX646856 | - | - | - |
|  | *Botryosphaeria agaves* | CBS 141505 | *Agave* sp. | MT592030 | - | - | - |
|  | ***Botryosphaeria dothidea*** | **CBS 115476** | *Prunus sp.* | AY236898 | - | - | - |
|  | *Botryosphaeria dothidea* | CBS 110302 | *Vitis vinifera* | AY573218 | - | - | - |
|  | *Botryosphaeria dothidea* | GZCC 160013 | Dead wood | KX447678 | - | - | - |
|  | *Botryosphaeria dothidea* | CGMCC 3.17723 | *Morus* sp. | KU221233 | - | - | - |
|  | *Botryosphaeria dothidea* | CGMCC 3.18744 | *Cedrus deodara* | KX278107 | - | - | - |
|  | ***Botryosphaeria fabicerciana*** | **CBS 127193** | *Eucalyptus* sp. | HQ332213 | - | - | - |
|  | *Botryosphaeria fabicerciana* | CBS 127194 | *Eucalyptus* sp. | HQ332214 | - | - | - |
|  | ***Botryosphaeria kuwatsukai*** | **CBS 135219** | *Malus domestica* | KJ433410 | - | - | - |
|  | *Botryosphaeria kuwatsukai* | CGMCC 3.18007 | *Malus* sp. | KX197094 | - | - | - |
|  | ***Botryosphaeria ramosa*** | **CBS 122069** | *Eucalyptus camaldulensis* | EU144070 | - | - | - |
|  | *Botryosphaeria ramosa* | CGMCC 3.18739 | *Eucalyptus* hybrid | KX278094 | - | - | - |
|  | *Botryosphaeria ramosa* | CGMCC 3.18741 | *Melastoma sanguineum* | KX278102 | - | - | - |
|  | ***Botryosphaeria scharifii*** | **CBS 124703** | *Mangifera indica* | JQ772057 | - | - | - |
|  | *Botryosphaeria scharifii* | CBS 124702 | *Mangifera indica* | JQ772056 | - | - | - |
| ***Diplodia*** | ***Diplodia allocellula*** | **CBS 130408** | *Acacia karroo* | JQ239384 | - | - | - |
|  | *Diplodia allocellula* | CBS 130410 | *Acacia karroo* | JQ239386 | - | - | - |
|  | ***Diplodia citricarpa*** | **CBS 124715** | *Citrus sp.* | KF890189 | - | - | - |
|  | *Diplodia citricarpa* | CBS 124714 | *Citrus sp.* | MT592039 | - | - | - |
|  | ***Diplodia eriobotrycola*** | **CBS 140851** | *Eriobotryia japonica* | MT592047 | - | - | - |
|  | *Diplodia eriobotrycola* | CPC 29680 | *Eriobotryia japonica* | KT240193 | - | - | - |
|  | *Diplodia estuarina* | CBS 139668 | *Aviccenia marina* | KP860676 | - | - | - |
|  | ***Diplodia pseudoseriata*** | **CBS 124906** | *Blepharocalyx salicifolius* | EU863181 | - | - | - |
|  | *Diplodia pseudoseriata* | UY1263 | *Myrciaria tenella* | EU863182 | - | - | - |
|  | *Diplodia pseudoseriata* | CBS 124933 | *Pterocarpus angolensis* | MT592065 | - | - | - |
|  | *Diplodia pseudoseriata* | CBS 124907 | *Hexachhlamis edulis* | MT592064 | - | - | - |
|  | *Diplodia pseudoseriata* | B45 | *Malus domestica* | KJ499680 | - | - | - |
|  | ***Diplodia sapinea*** | **CBS 393.84** | *Pinus nigra* | DQ458880 | - | - | - |
|  | *Diplodia sapinea* | CBS 121105 | *Prunus persica* | EF445377 | - | - | - |
|  | ***Diplodia scrobiculata*** | **CBS 118110** | *Pinus banksiana* | AY624253 | - | - | - |
|  | *Diplodia scrobiculata* | CBS 109944 | *Pinus greggii* | DQ458884 | - | - | - |
|  | ***Diplodia seriata*** | **CBS 112555** | *Quercus* sp. | AY573220 | - | - | - |
|  | *Diplodia seriata* | CBS 124139 | *Vitis vinifera* | GQ923848 | - | - | - |
| ***Neofusicoccum*** | ***Neofusicoccum australe*** | **CMW 6837** | *Acacia* sp. | AY339270 | - | - | - |
|  | *Neofusicoccum australe* | CMW 6853 | *Sequoiadendron giganteum* | AY339271 | - | - | - |
|  | ***Neofusicoccum cryptoaustrale*** | **CBS 122813** | *Eucalyptus* sp. | FJ752713 | - | - | - |
|  | *Neofusicoccum cryptoaustrale* | CMW 20738 | *Eeucalyptus citriodora* | FJ752710 | - | - | - |
|  | ***Neofusicoccum hongkongense*** | **CGMCC 3.18749** | *Araucaria cunninghamii* | KX278157 | - | - | - |
|  | *Neofusicoccum hongkongense* | CGMCC 3.18747 | *Araucaria cunninghamii* | KX278155 | - | - | - |
|  | ***Neofusicoccum kwambonambiense*** | **CBS 123639** | *Syzygium cordatum* | EU821870 | - | - | - |
|  | *Neofusicoccum kwambonambiense* | CBS 123641 | *Syzygium cordatum* | EU821889 | - | - | - |
|  | ***Neofusicoccum luteum*** | **CBS 110299** | *Vitis vinifera* | AY573217 | - | - | - |
|  | *Neofusicoccum luteum* | CBS 110497 | *Vitis vinifera* | EU673277 | - | - | - |
|  | *Neofusicoccum luteum* | **CMW 41365** | *Avicennia marina* | KP860702 | - | - | - |
|  | *Neofusicoccum luteum* | CMW 42481 | *Avicennia marina* | KP860692 | - | - | - |
|  | ***Neofusicoccum parvum*** | **ATCC 58191** | *Populus nigra* | AY236888 | - | - | - |
|  | *Neofusicoccum parvum* | CBS 110301 | *Vitis vinifera* | AY573221 | - | - | - |
|  | *Neofusicoccum parvum* | CDFA B139 | *Vaccinium corymbosum* | KJ126847 | - | - | - |
|  | *Neofusicoccum parvum* | CBS 145794 | *Rhaphiolepis indica* | MN175951 | - | - | - |
|  | ***Neofusicoccum ribis*** | **CBS 115475** | *Ribes* sp. | AY236877 | - | - | - |
|  | *Neofusicoccum ribis* | CBS 121.26 | *Ribes* sp. | AY236879 | - | - | - |
|  | ***Neofusicoccum stellenboschiana*** | **CBS 110864** | *Vitis vinifera* | AY343348 | - | - | - |
|  | ***Neofusicoccum vitifusiforme*** | **CBS 110887** | *Vitis vinifera* | AY343343 | - | - | - |
|  | *Neofusicoccum vitifusiforme* | CBS 121113 | *Prunus persica* | MT592254 | - | - | - |
| ***Dactylonectria*** | ***Dactylonectria alcacerensis*** | **CBS 129087** | *Vitis vinifera* | - | JF735630 | - | - |
|  | *Dactylonectria alcacerensis* | Cyl-01 | *Vitis vinifera* | - | MG745823 | - | - |
|  | *Dactylonectria alcacerensis* | Cy133 | *Vitis vinifera* | - | JF735628 | - | - |
|  | ***Dactylonectria ecuadoriensis*** | **MUCL55424** | *Piper* sp*.* | - | MF683683 | - | - |
|  | *Dactylonectria ecuadoriensis* | MUCL55425 | *Piper* sp. | - | MF683684 | - | - |
|  | ***Dactylonectria estremocencsis*** | **CBS 129085** | *Vitis vinifera* | - | JF735617 | - | - |
|  | *Dactylonectria estremocencsis* | Cy135 | *Vitis vinifera* | - | JF735615 | - | - |
|  | ***Dactylonectria hispanica*** | **CBS 142827** | *Pinus halepensis* | - | KY676864 | - | - |
|  | *Dactylonectria hispanica* | Cy228 | *Ficus* sp*.* | - | JF735578 | - | - |
|  | ***Dactylonectria macrodidyma*** | **CBS 112615** | *Vitis vinifera* | - | JF735647 | - | - |
|  | *Dactylonectria macrodidyma* | STE-U 9098 | *Olea europaea* subsp*. europaea* | - | MT309058 | - | - |
|  | *Dactylonectria macrodidyma* | Cy15UFSM | *Vitis vinifera* | - | KF633159 | - | - |
|  | ***Dactylonectria novozelandica*** | **CBS 113552** | *Vitis* sp. | - | JF735633 | - | - |
|  | *Dactylonectria novozelandica* | CBS 112608 | *Vitis vinifera* | - | JF735632 | - | - |
|  | *Dactylonectria novozelandica* | CBS 112593 | *Vitis vinifera* | - | JF735631 | - | - |
|  | ***Dactylonectria pauciseptata*** | **CBS 120171** | *Vitis* sp. | - | JF735587 | - | - |
|  | *Dactylonectria pauciseptata* | BV-1354 | *Vitis vinifera* | - | MK579256 | - | - |
|  | *Dactylonectria pauciseptata* | BV-1360 | *Vitis vinifera* | - | MK579258 | - | - |
|  | ***Dactylonectria pinicola*** | **CBS 173.37** | *Pinus laricio* | - | JF735614 | - | - |
|  | *Dactylonectria pinicola* | Cy200 | *Vitis vinifera* | - | JF735612 | - | - |
|  | ***Dactylonectria riojana*** | **BV-1396** | *Vitis* sp. | - | MK602831 | - | - |
|  | *Dactylonectria riojana* | BV-1397 | *Vitis* sp. | - | MK602832 | - | - |
|  | ***Dactylonectria torresensis*** | **CBS 129086** | *Vitis vinifera* | - | JF735681 | - | - |
|  | *Dactylonectria torresensis* | CBS 112598 | *Vitis vinifera* | - | JF735662 | - | - |
|  | *Dactylonectria torresensis* | CBS 188.49 | *Abies nordmanniana* | - | JF735658 | - | - |
|  | ***Dactylonectria valentina*** | **CBS 142826** | *Ilex aquifolium* | - | KY676863 | - | - |
|  | *Dactylonectria valentina* | STE-U 9105 | *Olea europaea* subsp*. europaea* | - | MT309072 | - | - |
| ***Ilyonectria*** | ***Ilyonectria crassa*** | **CBS 139.30** | *Lilium* sp*.* | - | JF735534 | - | - |
|  | *Ilyonectria crassa* | CBS 158.31 | *Narcissus* sp*.* | - | JF735535 | - | - |
|  | *Ilyonectria crassa* | CBS 129083 | *Panax quinquefolium* | - | JF735536 | - | - |
|  | ***Ilyonectria europea*** | **CBS 129078** | *Vitis vinifera* | - | JF735567 | - | - |
|  | *Ilyonectria europea* | CBS 102892 | Stem | - | JF735569 | - | - |
|  | ***Ilyonectria liriodendri*** | **CBS 110.81** | *Liriodedron tulipifera* | - | JF735507 | - | - |
|  | *Ilyonectria liriodendri* | CBS 112596 | *Vitis vinifera* | - | JF735511 | - | - |
|  | ***Ilyonectria lusitanica*** | **CBS 129080** | *Vitis vinifera* | - | JF735570 | - | - |
|  | ***Ilyonectria palmarum*** | **CBS 135754** | *Howea forsteriana* | - | HF922620 | - | - |
|  | *Ilyonectria palmarum* | CBS 135753 | *Howea forsteriana* | - | HF922621 | - | - |
|  | ***Ilyonectria panacis*** | **CBS 129079** | *Panax quinquefolium* | - | JF735572 | - | - |
|  | ***Ilyonectria pseudodestructans*** | **CBS 129081** | *Vitis vinifera* | - | JF735563 | - | - |
|  | *Ilyonectria pseudodestructans* | CBS 117824 | *Quercus* sp*.* | - | JF735562 | - | - |
|  | ***Ilyonectria rufa*** | **CBS 153.37** | *Dune sand* | - | JF735540 | - | - |
|  | *Ilyonectria rufa* | CBS 640.77 | *Abies alba* | - | JF735542 | - | - |
|  | ***Ilyonectria robusta*** | **CBS 308.35** | *Panax quinquefolium* | - | JF735518 | - | - |
|  | *Ilyonectria robusta* | Cy9UFSM | *Vitis vinifera* | - | KF633172 | - | - |
|  | ***Ilyonectria* sp*.*** | **8918** | *Prunus persica* sp. | - | MK765799 | - | - |
| ***Phaeoacremonium*** | ***Phaeoacremonium angustius*** | **CBS 114992** | *Vitis vinifera* | - | - | DQ173104 | - |
|  | *Phaeoacremonium angustius* | CBS 114991 | *Vitis vinifera* | - | - | DQ173103 | - |
|  | ***Phaeoacremonium argentinense*** | **CBS 777.83** | Soil | - | - | DQ173108 | - |
|  | ***Phaeoacremonium austroafricanum*** | **CBS 112949** | *Vitis vinifera* | - | - | DQ173099 | - |
|  | *Phaeoacremonium austroafricanum* | CBS 114994 | *Vitis vinifera* | - | - | DQ173102 | - |
|  | ***Phaeoacremonium fraxinopennsylvanicum*** | **CBS 101585** | *Vitis vinifera* | - | - | KF764684 | - |
|  | *Phaeoacremonium fraxinopennsylvanicum* | STE-U 6987 | *Vitis vinifera* | - | - | JQ038913 | - |
|  | ***Phaeoacremonium globosum*** | **ICMP16988** | *Vitis berlandieri* × *Vitis riparia* | - | - | EU596525 | - |
|  | *Phaeoacremonium globosum* | ICMP16987 | *Vitis berlandieri* × *Vitis riparia* | - | - | EU596527 | - |
|  | ***Phaeoacremonium iranium*** | **CBS 101357** | *Vitis vinifera* | - | - | DQ173097 | - |
|  | *Phaeoacremonium iranium* | CBS 117114 | *Vitis vinifera* | - | - | DQ173098 | - |
|  | ***Phaeoacremonium longicollarum*** | **CBS 142699** | *Prunus armeniaca* | - | - | KY906689 | - |
|  | *Phaeoacremonium longicollarum* | CBS 142700 | *Psidium guajava* | - | - | KY906879 | - |
|  | ***Phaeoacremonium minimum*** | **CBS 246.91** | *Vitis vinifera* | - | - | AF246811 | - |
|  | *Phaeoacremonium minimum* | CBS 110703 | *Vitis vinifera* | - | - | DQ173094 | - |
|  | *Phaeoacremonium minimum* | Pm-06 | *Vitis vinifera* | - | - | MG745808 | - |
|  | ***Phaeoacremonium oleae*** | **CBS 142704** | *Olea europaea* | - | - | KY906937 | - |
|  | *Phaeoacremonium oleae* | CBS 142703 | *Olea europaea* | - | - | KY906891 | - |
|  | *Phaeoacremonium pseudopanacis* | CBS 142101 | *Pseudopanax crassifolius* | - | - | KY173609 | - |
|  | ***Phaeoacremonium tectonae*** | **MFLUCC 13-0707** | *Tectona grandis* | - | - | KT285563 | - |
|  | *Phaeoacremonium tectonae* | MFLUCC 14-1125 | *Tectona grandis* | - | - | KT285565 | - |
|  | ***Phaeoacremonium viticola*** | **CBS 101738** | *Vitis vinifera* | - | - | AF192391 | - |
|  | *Phaeoacremonium viticola* | CBS 101737 | *Vitis vinifera* | - | - | AF246817 | - |
| **Phaeomoniellales** | ***Aequabiliella effusa*** | **CBS 120883** | *Prunus salicina* | - | - | - | NR132005 |
|  | *Celeriorella umnqumae* | STE-U 8442 | *Olea europaea* | - | - | - | MT791052 |
|  | ***Celerioriella dura*** | **CBS 120882** | *Prunus salicina* | - | - | - | NR132004 |
|  | ***Celerioriella prunicola*** | **CBS 120876** | *Prunus salicina* | - | - | - | NR132003 |
|  | ***Celerioriella umnqumae*** | **CBS 146756** | *Olea europaea* | - | - | - | MT791051 |
|  | *Celerioriella umnqumae* | STE-U 8442 | *Olea europaea* | - | - | - | MT791052 |
|  | ***Paraphaeoisaria alabamensis*** | **CBS 101.77B** | *Cronartium quercuum* | - | - | - | MH861029 |
|  | *Paraphaeoisaria alabamensis* | CBS 101.77A | *Cronartium quercuum* | - | - | - | MH861028 |
|  | ***Paraphaeomoniella capensis*** | **CBS 123535** | *Encephalartos altensteinii* | - | - | - | NR137711 |
|  | ***Paraphaeomoniella pinifoliorum*** | **CBS 114903** | *Vitis vinifera* | - | - | - | NR160218 |
|  | ***Phaeomoniella chlamydospora*** | **CBS 229.95** | *Vitis vinifera* | - | - | - | NR155612 |
|  | *Phaeomoniella chlamydospora* | STEU7538 | *Vitis vinifera* | - | - | - | KU244284 |
|  | *Phaeomoniella chlamydospora* | CBS 145016 | *Vitis vinifera* | - | - | - | MH999525 |
|  | ***Pseudophaeomoniella globosa*** | **CBS 146755** | *Olea europaea* | - | - | - | MT791056 |
|  | *Pseudophaeomoniella globosa* | STE-U 7946 | *Olea europaea* | - | - | - | MT791062 |
|  | ***Pseudophaeomoniella oleae*** | **CBS 139191** | *Olea europaea* | - | - | - | NR137966 |
|  | ***Pseudophaeomoniella oleicola*** | **CBS 139192** | *Olea europaea* | - | - | - | NR137965 |
|  | *Pseudophaeomoniella oleicola* | STE-U 7933 | *Olea europaea* | - | - | - | MW008603 |
|  | ***Vredendaliella oleae*** | **CBS 146757** | *Olea europaea* | - | - | - | MT791073 |
|  | *Xenocylindrosporium* sp. CFJS2015c | STE-U 8436 | *Olea europaea* | - | - | - | MT791080 |
|  | *Xenocylindrosporium* sp. CFJS2015c | CSN 1180 | *Olea europaea* | - | - | - | MT791077 |
|  | *Xenocylindrosporium* sp. CFJS2015e | STE-U 8438 | *Olea europaea* | - | - | - | MT791079 |
| ***Diaporthe*** | ***Diaporthe ambigua*** | **CBS 114015** | *Pyrus communis* | KC343736 | - | - | - |
|  | *Diaporthe ambigua* | CBS 117167 | *Aspalathus linearis* | KC343737 | - | - | - |
|  | ***Diaporthe ampelina*** | **CBS 114016** | *Vitis vinifera* | GQ250351 | - | - | - |
|  | *Diaporthe ampelina* | CBS 111888 | *Vitis vinifera* | KC343742 | - | - | - |
|  | *Diaporthe ampelina* | CPC 29674 | *Vitis vinifera* | MG281514 | - | - | - |
|  | *Diaporthe ampelina* | EFA 460 | *Vitis vinifera* | MH051278 | - | - | - |
|  | ***Diaporthe amygdali*** | **CBS 126679** | *Prunus dulcis* | KC343748 | - | - | - |
|  | *Diaporthe amygdali* | CBS 111811 | *Vitis vinifera* | KC343745 | - | - | - |
|  | ***Diaporthe anacardii*** | **CBS 720.97** | *Anacardium occidentale* | KC343750 | - | - | - |
|  | ***Diaporthe baccae*** | **CBS 136972** | *Vaccinium corymbosum* | KJ160597 | - | - | - |
|  | *Diaporthe baccae* | CBS 143343 | *Vitis vinifera* | MG281522 | - | - | - |
|  | ***Diaporthe bohemiae*** | **CBS 143347** | *Vitis* spp. | MG281536 | - | - | - |
|  | *Diaporthe bohemiae* | CBS 143348 | *Vitis* spp. | MG281537 | - | - | - |
|  | ***Diaporthe celastrina*** | **CBS 139.27** | *Celastrus* sp. | KC343773 | - | - | - |
|  | ***Diaporthe celeris*** | **CBS 143349** | *Vitis vinifera* | MG281538 | - | - | - |
|  | *Diaporthe celeris* | CPC 28267 | *Vitis vinifera* | MG281540 | - | - | - |
|  | ***Diaporthe eres*** | **CBS 138594** | *Ulmus laevis* | KJ210550 | - | - | - |
|  | *Diaporthe eres* | CPC 28226 | *Vitis vinifera* | MG281546 | - | - | - |
|  | *Diaporthe fibrosa* | CBS 109751 | *Rhamnus cathartica* | KC343825 | - | - | - |
|  | ***Diaporthe foeniculina*** | **CBS 111553** | *Foeniculum vulgare* | KC343827 | - | - | - |
|  | *Diaporthe foeniculina* | CBS 187.27 | *Camellia sinensis* | KC343833 | - | - | - |
|  | ***Diaporthe helianthi*** | **CBS 592.81** | *Helianthus annuus* | KC343841 | - | - | - |
|  | ***Diaporthe helicis*** | **CBS 138596** | *Hedera helix* | KJ210559 | - | - | - |
|  | ***Diaporthe hispaniae*** | **CBS 143351** | *Vitis vinifera* | MG281644 | - | - | - |
|  | *Diaporthe hispaniae* | CBS 143352 | *Vitis vinifera* | MG281645 | - | - | - |
|  | ***Diaporthe hungariae*** | **CBS 143353** | *Vitis vinifera* | MG281647 | - | - | - |
|  | *Diaporthe hungariae* | CBS 143354 | *Vitis vinifera* | MG281648 | - | - | - |
|  | *Diaporthe phaseolorum* | CBS 113425 | *Olearia* cf. *rani* | KC343900 | - | - | - |
|  | *Diaporthe phaseolorum* | CBS 127465 | *Actinidia chinensis* | KC343903 | - | - | - |
|  | ***Diaporthe pulla*** | **CBS 338.89** | *Hedera helix* | KC343878 | - | - | - |
|  | ***Diaporthe rudis*** | **CBS 109292** | *Laburnum anagyroides* | KC843090 | - | - | - |
|  | *Diaporthe rudis* | CBS 113201 | *Vitis vinifera* | KC343960 | - | - | - |
|  | ***Diaporthe schini*** | **CBS 133181** | *Schinus terebinthifolius* | KC343917 | - | - | - |
|  | ***Diaporthe sojae*** | **CBS 139282** | *Glycine max* | KJ590762 | - | - | - |
|  | *Diaporthe sojae* | CBS 116019 | *Caperonia palustris* | KC343901 | - | - | - |
|  | ***Diaporthe terebinthifolii*** | **CBS 133180** | *Schinus terebinthifolius* | KC343942 | - | - | - |
| **Outgroups** | *Calosphaeria africana* | CBS 120870 | *Prunus armeniaca* | - | - | EU367464 | - |
|  | ***Campylocarpon fasciculare*** | **CBS 112613** | *Vitis vinifera* | - | JF735502 | - | - |
|  | ***Campylocarpon pseudofasciculare*** | **CBS 112679** | *Vitis vinifera* | - | JF735503 | - | - |
|  | ***Cosmospora arxii*** | **CBS 748.69** | *Hypoxylon* sp. | - | - | - | NR145062- |
|  | ***Cosmospora butry*** | CBS 301.308 | Butter | - | - | - | MW827605 |
|  | ***Diaporthella corylina*** | CBS 121124 | *Corylus* sp. | KC343730 | - | - | - |
|  | ***Diaporthella cryptica*** | **CBS 140348** | *Corylus avellana* | MN271854 | - | - | - |
|  | *Jattaea algeriensis* | CBS 120871 | *Prunus salicina* | - | - | EU367466 | - |
|  | ***Lasiodiplodia theobromae*** | **CBS 164.96** | Fruit along coral reef coast | AY640258 | - | - | - |
|  | ***Lasiodiploida parva*** | **CBS 456.78** | Cassava-field-soil | EF622063 | - | - | - |

**Supplementary Table 2.** Evolutionary models of nucleotide substitution used for the Bayesian Inference analysis, suggested by jModelTest2 according to the Akaike information criterion (AIC).

| **Taxon** | **AIC^1^** |
| --- | --- |
| *Botryosphaeria* | GTR + G |
| *Diplodia* | GTR + G |
| *Neofusicoccum* | HKY85 + I + G |
| *Dactylonectria* | GTR + G |
| *Ilyonectria* | GTR + G |
| *Phaeoacremonium* | GTR + G |
| *Phaeomoniella* | GTR + I |
| *Diaporthe* | HKY85 + I + G |

^1^ GTR = Generalised Time Reversible model

HKY85 = Hasegawa-Kishino-Yano 85 model

I = refers to proportion of invariable sites estimated

G = refers to gamma distribution parameter estimated

**Supplementary Table 3.** Uruguayan isolates of grapevine trunk diseases pathogens obtained from nursery propagation materials of different grapevine cultivars and rootstocks

| **Fungal species** | **Isolate** | **Cultivar/Rootstock** | **Symptomatic or asymptomatic material** | **Nursery propagation stage^1^** | **Year of collection** | **Gene region^2^** | **GenBank accesion no.** |  |
| --- | --- | --- | --- | --- | --- | --- | --- | --- |
| *Botryosphaeria dothidea* | URU-VD-1 | Gravesac | symptomatic | 2 | 2018 | TEF | ON573060 |  |
|  | URU-VD-2 | Merlot | asymptomatic | 1 | 2018 | TEF | ON573061 |  |
|  | URU-VD-3 | 3309C | symptomatic | 1 | 2018 | TEF | ON573062 |  |
|  | URU-VD-4 | Albariño | symptomatic | 1 | 2018 | TEF | ON573063 |  |
|  | URU-VD-5 | Tannat/Gravesac | symptomatic | 4 | 2018 | TEF | ON573064 |  |
|  | URU-VD-6 | Cabernet Franc/3309C | symptomatic | 4 | 2018 | TEF | ON573065 |  |
|  | URU-VD-7 | Chardonnay/SO4 | symptomatic | 4 | 2018 | TEF | ON573066 |  |
|  | URU-VD-8 | Marselan | asymptomatic | 1 | 2019 | TEF | ON573067 |  |
|  | URU-VD-9 | Gravesac | asymptomatic | 1 | 2019 | TEF | ON573068 |  |
|  | URU-VD-10 | 3309C | asymptomatic | 1 | 2019 | TEF | ON573069 |  |
|  | URU-VD-11 | Albariño/Gravesac | symptomatic | 4 | 2019 | TEF | ON573070 |  |
|  | URU-VD-12 | Albariño/Gravesac | symptomatic | 4 | 2019 | TEF | ON573071 |  |
|  | URU-VD-13 | Tannat/1103P | symptomatic | 4 | 2019 | TEF | ON573072 |  |
|  | URU-VD-14 | Tannat/1103P | symptomatic | 4 | 2019 | TEF | ON573073 |  |
|  | URU-VD-15 | Tannat | symptomatic | 2 | 2019 | TEF | ON573074 |  |
|  | URU-VD-16 | Albariño/SO4 | symptomatic | 3 | 2019 | TEF | ON573075 |  |
| *Diplodia pseudoseriata* | URU-VD-18 | Merlot | asymptomatic | 2 | 2018 | TEF | ON573076 |  |
|  | URU-VD-19 | Merlot | symptomatic | 2 | 2018 | TEF | ON573077 |  |
|  | URU-VD-20 | Gravesac | asymptomatic | 2 | 2018 | TEF | ON573078 |  |
|  | URU-VD-21 | 1103P | symptomatic | 2 | 2018 | TEF | ON573079 |  |
|  | URU-VD-22 | 101-14 | asymptomatic | 1 | 2018 | TEF | ON573080 |  |
|  | URU-VD-24 | 101-14 | asymptomatic | 2 | 2018 | TEF | ON573081 |  |
|  | URU-VD-25 | Tannat | asymptomatic | 1 | 2019 | TEF | ON573082 |  |
| *Diplodia seriata* | URU-VD-17 | Merlot | asymptomatic | 2 | 2018 | TEF | ON573083 |  |
|  | URU-VD-23 | Lácrima-Christi | asymptomatic | 2 | 2018 | TEF | ON573084 |  |
| *Neofusicoccum australe* | URU-VD-27 | Merlot | symptomatic | 2 | 2018 | TEF | ON573085 |  |
| *Neofusicoccum cryptoaustrale* | URU-VD-28 | Gravesac | asymptomatic | 1 | 2018 | TEF | ON573086 |  |
|  | URU-VD-31 | Albariño | asymptomatic | 2 | 2018 | TEF | ON573087 |  |
|  | URU-VD-36 | Albariño/Gravesac | symptomatic | 3 | 2018 | TEF | ON573088 |  |
| *Neofusicoccum luteum* | URU-VD-30 | Albariño | asymptomatic | 1 | 2018 | TEF | ON573089 |  |
|  | URU-VD-37 | Lácrima/1103P | asymptomatic | 4 | 2019 | TEF | ON573090 |  |
|  | URU-VD-39 | Tannat/1103P | symptomatic | 4 | 2019 | TEF | ON573091 |  |
| *Neofusicoccum parvum* | URU-VD-26 | 1103P | asymptomatic | 2 | 2018 | TEF | ON573092 |  |
|  | URU-VD-29 | Lácrima-Christi | symptomatic | 1 | 2018 | TEF | ON573093 |  |
|  | URU-VD-32 | Moscatel de Hamburgo/SO4 | asymptomatic | 3 | 2018 | TEF | ON573094 |  |
|  | URU-VD-33 | Lácrima-Christi/SO4 | asymptomatic | 3 | 2018 | TEF | ON573095 |  |
|  | URU-VD-34 | Merlot/101-14 | symptomatic | 4 | 2018 | TEF | ON573096 |  |
|  | URU-VD-35 | Albariño/101-14 | asymptomatic | 3 | 2019 | TEF | ON573097 |  |
|  | URU-VD-38 | Albariño/101-14 | symptomatic | 4 | 2019 | TEF | ON573098 |  |
|  | URU-VD-40 | 3309C | symptomatic | 2 | 2019 | TEF | ON573099 |  |
|  | URU-VD-41 | Albariño/SO4 | asymptomatic | 3 | 2019 | TEF | ON573100 |  |
|  | URU-VD-42 | Tannat/Gravesac | symptomatic | 3 | 2019 | TEF | ON573101 |  |
|  | URU-VD-43 | Tannat/Gravesac | symptomatic | 3 | 2019 | TEF | ON573102 |  |
|  | URU-VD-44 | Marselan/3309C | symptomatic | 3 | 2019 | TEF | ON573103 |  |
|  | URU-VD-45 | Albariño/101-14 | symptomatic | 4 | 2019 | TEF | ON573104 |  |
|  | URU-VD-46 | Tannat | symptomatic | 2 | 2019 | TEF | ON573105 |  |
| *Dactylonectria macrodidyma* | URU-VD-47 | Merlot/101-14 | symptomatic | 4 | 2018 | HIS3 | ON573132 |  |
|  | URU-VD-49 | Merlot/101-14 | symptomatic | 4 | 2018 | HIS3 | ON573133 |  |
|  | URU-VD-51 | Merlot/101-14 | symptomatic | 4 | 2018 | HIS3 | ON573134 |  |
|  | URU-VD-66 | Tannat/1103P | symptomatic | 4 | 2019 | HIS3 | ON573135 |  |
|  | URU-VD-73 | Albariño/101-14 | symptomatic | 4 | 2019 | HIS3 | ON573136 |  |
|  | URU-VD-75 | Albariño/Gravesac | symptomatic | 4 | 2019 | HIS3 | ON573137 |  |
|  | URU-VD-77 | Albariño/Gravesac | symptomatic | 4 | 2019 | HIS3 | ON573138 |  |
|  | URU-VD-78 | Albariño/Gravesac | symptomatic | 4 | 2019 | HIS3 | ON573139 |  |
|  | URU-VD-80 | Tannat/1103P | symptomatic | 4 | 2019 | HIS3 | ON573140 |  |
|  | URU-VD-81 | Tannat/1103P | symptomatic | 4 | 2019 | HIS3 | ON573141 |  |
|  | URU-VD-82 | Tannat/1103P | symptomatic | 4 | 2019 | HIS3 | ON573142 |  |
| *Dactylonectria novozelandica* | URU-VD-58 | Albariño/101-14 | symptomatic | 4 | 2019 | HIS3 | ON573143 |  |
|  | URU-VD-60 | Albariño/101-14 | symptomatic | 4 | 2019 | HIS3 | ON573144 |  |
|  | URU-VD-61 | Albariño/101-14 | symptomatic | 4 | 2019 | HIS3 | ON573145 |  |
|  | URU-VD-62 | Albariño/101-14 | symptomatic | 4 | 2019 | HIS3 | ON573146 |  |
|  | URU-VD-63 | Albariño/101-14 | symptomatic | 4 | 2019 | HIS3 | ON573147 |  |
|  | URU-VD-64 | Albariño/Gravesac | symptomatic | 4 | 2019 | HIS3 | ON573148 |  |
|  | URU-VD-65 | Albariño/Gravesac | symptomatic | 4 | 2019 | HIS3 | ON573149 |  |
|  | URU-VD-67 | Tannat/1103P | symptomatic | 4 | 2019 | HIS3 | ON573150 |  |
|  | URU-VD-68 | Tannat/1103P | symptomatic | 4 | 2019 | HIS3 | ON573151 |  |
|  | URU-VD-69 | Tannat/1103P | symptomatic | 4 | 2019 | HIS3 | ON573152 |  |
|  | URU-VD-70 | Albariño/101-14 | symptomatic | 4 | 2019 | HIS3 | ON573153 |  |
|  | URU-VD-71 | Albariño/101-14 | symptomatic | 4 | 2019 | HIS3 | ON573154 |  |
|  | URU-VD-72 | Albariño/101-14 | symptomatic | 4 | 2019 | HIS3 | ON573155 |  |
|  | URU-VD-74 | Albariño/101-14 | symptomatic | 4 | 2019 | HIS3 | ON573156 |  |
|  | URU-VD-76 | Albariño/Gravesac | symptomatic | 4 | 2019 | HIS3 | ON573157 |  |
| *Dactylonectria pauciseptata* | URU-VD-52 | Tannat/Gravesac | symptomatic | 4 | 2018 | HIS3 | ON573158 |  |
|  | URU-VD-54 | Chardonnay/SO4 | symptomatic | 4 | 2018 | HIS3 | ON573159 |  |
|  | URU-VD-59 | Tannat/1103P | symptomatic | 4 | 2019 | HIS3 | ON573160 |  |
| *Dactylonectria torresensis* | URU-VD-48 | Merlot/101-14 | symptomatic | 4 | 2018 | HIS3 | ON573161 |  |
|  | URU-VD-50 | Merlot/101-14 | symptomatic | 4 | 2018 | HIS3 | ON573162 |  |
|  | URU-VD-53 | Tannat/Gravesac | symptomatic | 4 | 2018 | HIS3 | ON573163 |  |
|  | URU-VD-55 | Chardonnay/SO4 | symptomatic | 4 | 2018 | HIS3 | ON573164 |  |
|  | URU-VD-56 | Lácrima/1103P | symptomatic | 4 | 2018 | HIS3 | ON573165 |  |
|  | URU-VD-79 | Albariño/Gravesac | symptomatic | 4 | 2019 | HIS3 | ON573166 |  |
| *Dactylonectria valentina* | URU-VD-57 | Lácrima/1103P | symptomatic | 4 | 2018 | HIS3 | ON573167 |  |
| *Ilyonectria liriodendri* | URU-VD-85 | Tannat/Gravesac | symptomatic | 4 | 2018 | HIS3 | ON573168 |  |
|  | URU-VD-86 | Albariño/Gravesac | symptomatic | 4 | 2019 | HIS3 | ON573169 |  |
|  | URU-VD-87 | Albariño/Gravesac | symptomatic | 4 | 2019 | HIS3 | ON573170 |  |
|  | URU-VD-88 | Tannat/1103P | symptomatic | 4 | 2019 | HIS3 | ON573171 |  |
| *Ilyonectria robusta* | URU-VD-83 | Tannat/Gravesac | symptomatic | 4 | 2018 | HIS3 | ON573172 |  |
| *Ilyonectria* sp. | URU-VD-84 | Tannat/Gravesac | symptomatic | 4 | 2018 | HIS3 | ON573173 |  |
| *Phaeoacremonium minimum* | URU-VD-89 | Merlot/101-14 | symptomatic | 4 | 2018 | TUB2 | ON573174 |  |
|  | URU-VD-90 | Merlot/101-14 | symptomatic | 4 | 2018 | TUB2 | ON573175 |  |
|  | URU-VD-91 | Tannat/Gravesac | symptomatic | 4 | 2018 | TUB2 | ON573176 |  |
|  | URU-VD-92 | Tannat/Gravesac | symptomatic | 4 | 2018 | TUB2 | ON573177 |  |
|  | URU-VD-93 | Tannat/Gravesac | symptomatic | 4 | 2018 | TUB2 | ON573178 |  |
|  | URU-VD-94 | Tannat/Gravesac | symptomatic | 4 | 2018 | TUB2 | ON573179 |  |
|  | URU-VD-95 | Tannat/Gravesac | symptomatic | 4 | 2018 | TUB2 | ON573180 |  |
|  | URU-VD-96 | Tannat/Gravesac | symptomatic | 4 | 2018 | TUB2 | ON573181 |  |
|  | URU-VD-97 | Tannat/Gravesac | symptomatic | 4 | 2018 | TUB2 | ON573182 |  |
|  | URU-VD-98 | Tannat/Gravesac | symptomatic | 4 | 2018 | TUB2 | ON573183 |  |
|  | URU-VD-99 | Chardonnay/SO4 | symptomatic | 4 | 2018 | TUB2 | ON573184 |  |
|  | URU-VD-100 | Chardonnay/SO4 | symptomatic | 4 | 2018 | TUB2 | ON573185 |  |
|  | URU-VD-101 | Tannat/Gravesac | symptomatic | 4 | 2018 | TUB2 | ON573186 |  |
|  | URU-VD-102 | Tannat/Gravesac | symptomatic | 4 | 2018 | TUB2 | ON573187 |  |
|  | URU-VD-103 | Chardonnay/SO4 | symptomatic | 4 | 2018 | TUB2 | ON573188 |  |
|  | URU-VD-104 | Chardonnay/SO4 | symptomatic | 4 | 2018 | TUB2 | ON573189 |  |
|  | URU-VD-106 | Chardonnay/SO4 | symptomatic | 4 | 2018 | TUB2 | ON573190 |  |
|  | URU-VD-107 | Chardonnay/SO4 | symptomatic | 4 | 2018 | TUB2 | ON573191 |  |
|  | URU-VD-108 | Chardonnay/SO4 | symptomatic | 4 | 2018 | TUB2 | ON573192 |  |
|  | URU-VD-109 | Chardonnay/SO4 | symptomatic | 4 | 2018 | TUB2 | ON573193 |  |
|  | URU-VD-110 | Lácrima Christi/1103P | symptomatic | 4 | 2018 | TUB2 | ON573194 |  |
|  | URU-VD-111 | Lácrima Christi/1103P | symptomatic | 4 | 2018 | TUB2 | ON573195 |  |
|  | URU-VD-112 | Lácrima Christi/1103P | symptomatic | 4 | 2018 | TUB2 | ON573196 |  |
|  | URU-VD-113 | Lácrima Christi/1103P | symptomatic | 4 | 2018 | TUB2 | ON573197 |  |
|  | URU-VD-114 | Lácrima Christi/1103P | symptomatic | 4 | 2018 | TUB2 | ON573198 |  |
|  | URU-VD-116 | Lácrima Christi/1103P | symptomatic | 4 | 2018 | TUB2 | ON573199 |  |
|  | URU-VD-117 | Albariño/101-14 | symptomatic | 4 | 2019 | TUB2 | ON573200 |  |
|  | URU-VD-118 | Albariño/101-14 | symptomatic | 4 | 2019 | TUB2 | ON573201 |  |
|  | URU-VD-119 | Albariño/101-14 | symptomatic | 4 | 2019 | TUB2 | ON573202 |  |
|  | URU-VD-120 | Albariño/101-14 | symptomatic | 4 | 2019 | TUB2 | ON573203 |  |
|  | URU-VD-121 | Albariño/Gravesac | symptomatic | 4 | 2019 | TUB2 | ON573204 |  |
|  | URU-VD-122 | Tannat/1103P | symptomatic | 4 | 2019 | TUB2 | ON573205 |  |
|  | URU-VD-123 | Tannat/1103P | symptomatic | 4 | 2019 | TUB2 | ON573206 |  |
|  | URU-VD-124 | Tannat/1103P | symptomatic | 4 | 2019 | TUB2 | ON573207 |  |
|  | URU-VD-125 | Tannat/1103P | symptomatic | 4 | 2019 | TUB2 | ON573208 |  |
|  | URU-VD-126 | Tannat/1103P | symptomatic | 4 | 2019 | TUB2 | ON573209 |  |
|  | URU-VD-127 | Tannat/1103P | symptomatic | 4 | 2019 | TUB2 | ON573210 |  |
|  | URU-VD-128 | Tannat/1103P | symptomatic | 4 | 2019 | TUB2 | ON573211 |  |
|  | URU-VD-129 | Tannat/1103P | symptomatic | 4 | 2019 | TUB2 | ON573212 |  |
|  | URU-VD-130 | Tannat/Gravesac | symptomatic | 3 | 2019 | TUB2 | ON573213 |  |
|  | URU-VD-131 | Marselan/3309C | symptomatic | 3 | 2019 | TUB2 | ON573214 |  |
| *Phaeoacremonium austroafricanum* | URU-VD-105 | Chardonnay/SO4 | symptomatic | 4 | 2018 | TUB2 | ON573215 |  |
| *Phaeoacremonium oleae* | URU-VD-115 | Lácrima Christi/1103P | asymptomatic | 4 | 2018 | TUB2 | ON573216 |  |
| *Phaeomoniella chlamydospora* | URU-VD-132 | Gravesac | symptomatic | 1 | 2018 | ITS | ON584361 |  |
|  | URU-VD-133 | 1103P | asymptomatic | 1 | 2018 | ITS | ON584362 |  |
|  | URU-VD-134 | Marselan | symptomatic | 1 | 2018 | ITS | ON584363 |  |
|  | URU-VD-135 | Moscatel de Hamburgo/SO4 | asymptomatic | 3 | 2018 | ITS | ON584364 |  |
|  | URU-VD-136 | Cabernet Franc/3309C | symptomatic | 4 | 2018 | ITS | ON584365 |  |
|  | URU-VD-137 | Cabernet Franc/3309C | symptomatic | 4 | 2018 | ITS | ON584366 |  |
|  | URU-VD-138 | Cabernet Franc/3309C | symptomatic | 4 | 2018 | ITS | ON584367 |  |
|  | URU-VD-139 | 3309C | asymptomatic | 1 | 2018 | ITS | ON584368 |  |
|  | URU-VD-140 | Tannat/Gravesac | symptomatic | 4 | 2018 | ITS | ON584369 |  |
|  | URU-VD-141 | Chardonnay/SO4 | symptomatic | 4 | 2018 | ITS | ON584370 |  |
|  | URU-VD-142 | Chardonnay/SO4 | symptomatic | 4 | 2018 | ITS | ON584371 |  |
|  | URU-VD-143 | Marselan | symptomatic | 1 | 2019 | ITS | ON584372 |  |
|  | URU-VD-144 | Albariño/101-14 | symptomatic | 4 | 2019 | ITS | ON584373 |  |
|  | URU-VD-145 | Albariño/101-14 | symptomatic | 4 | 2019 | ITS | ON584374 |  |
|  | URU-VD-146 | Albariño/101-14 | symptomatic | 4 | 2019 | ITS | ON584375 |  |
|  | URU-VD-147 | Albariño/101-14 | symptomatic | 4 | 2019 | ITS | ON584376 |  |
|  | URU-VD-148 | Albariño/101-14 | symptomatic | 4 | 2019 | ITS | ON584377 |  |
|  | URU-VD-149 | Albariño/101-14 | symptomatic | 4 | 2019 | ITS | ON584378 |  |
|  | URU-VD-150 | Albariño/Gravesac | symptomatic | 4 | 2019 | ITS | ON584379 |  |
|  | URU-VD-151 | Albariño/Gravesac | symptomatic | 4 | 2019 | ITS | ON584380 |  |
|  | URU-VD-152 | Tannat/1103P | symptomatic | 4 | 2019 | ITS | ON584381 |  |
|  | URU-VD-153 | Tannat/1103P | symptomatic | 4 | 2019 | ITS | ON584382 |  |
|  | URU-VD-154 | Marselan/3309C | symptomatic | 3 | 2019 | ITS | ON584383 |  |
| *Diaporthe baccae* | URU-VD-160 | Albariño | asymptomatic | 2 | 2018 | TEF | ON573106 |  |
|  | URU-VD-161 | Albariño | symptomatic | 2 | 2018 | TEF | ON573107 |  |
| *Diaporthe eres* | URU-VD-171 | Chardonnay/SO4 | symptomatic | 4 | 2018 | TEF | ON573108 |  |
|  | URU-VD-172 | Chardonnay/SO4 | symptomatic | 4 | 2018 | TEF | ON573109 |  |
| *Diaporthe foeniculina* | URU-VD-177 | Tannat | symptomatic | 1 | 2019 | TEF | ON573110 |  |
|  | URU-VD-178 | Albariño/101-14 | symptomatic | 4 | 2019 | TEF | ON573111 |  |
| *Diaporthe* sp. | URU-VD-155 | 3309C | symptomatic | 1 | 2018 | TEF | ON573112 |  |
|  | URU-VD-156 | Tannat | asymptomatic | 1 | 2018 | TEF | ON573113 |  |
|  | URU-VD-157 | Marselan | asymptomatic | 2 | 2018 | TEF | ON573114 |  |
|  | URU-VD-158 | Marselan | asymptomatic | 2 | 2018 | TEF | ON573115 |  |
|  | URU-VD-159 | Marselan | symptomatic | 2 | 2018 | TEF | ON573116 |  |
|  | URU-VD-162 | Moscatel de Hamburgo/SO4 | asymptomatic | 3 | 2018 | TEF | ON573117 |  |
|  | URU-VD-163 | Tannat/Gravesac | symptomatic | 4 | 2018 | TEF | ON573118 |  |
|  | URU-VD-164 | Moscatel de Hamburgo/SO4 | asymptomatic | 3 | 2018 | TEF | ON573119 |  |
|  | URU-VD-165 | Cabernet Franc/3309C | symptomatic | 4 | 2018 | TEF | ON573120 |  |
|  | URU-VD-166 | Merlot/101-14 | symptomatic | 4 | 2018 | TEF | ON573121 |  |
|  | URU-VD-167 | Merlot/101-14 | symptomatic | 4 | 2018 | TEF | ON573122 |  |
|  | URU-VD-168 | Tannat/Gravesac | symptomatic | 4 | 2018 | TEF | ON573123 |  |
|  | URU-VD-169 | Tannat/Gravesac | symptomatic | 4 | 2018 | TEF | ON573124 |  |
|  | URU-VD-170 | Tannat/Gravesac | symptomatic | 4 | 2018 | TEF | ON573125 |  |
|  | URU-VD-173 | Tannat | asymptomatic | 1 | 2019 | TEF | ON573126 |  |
|  | URU-VD-174 | Marselan | asymptomatic | 1 | 2019 | TEF | ON573127 |  |
|  | URU-VD-175 | Marselan | symptomatic | 1 | 2019 | TEF | ON573128 |  |
|  | URU-VD-179 | Albariño/101-14 | symptomatic | 4 | 2019 | TEF | ON573130 |  |
|  | URU-VD-180 | Tannat/Gravesac | symptomatic | 3 | 2019 | TEF | ON573131 |  |
| *Diaporthe terebinthifolii* | URU-VD-176 | Marselan | asymptomatic | 1 | 2019 | TEF | ON573129 |  |
| ^1^ Stage 1 = rootstocks and scion cuttings from mother plants; Stage 2 = rootstocks and scion cuttings after cold storage and hydration; Stage 3 = grafted plants after callusing; Stage 4= rooted grafted plants ready to plant | | | | | | | | |

^2^ TEF = elongation factor 1-α; HIS3 = histone 3; TUB2 = beta-tubulin; ITS = internal transcribed spacer region and 5.8S rRNA
